# Crystal structures of (±)-(1*SR*,5*SR*,6*SR*,7*SR*,10*SR*,11*SR*,13*RS*,14*SR*)-13-hy­droxy-7-meth­oxy­meth­oxy-11,15,18,18-tetra­methyl-3-oxo-2,4-dioxa­tetra­cyclo­[12.3.1.0^1,5^.0^6,11^]octa­dec-15-en-10-yl benzoate, its 13-epimer and 13-one derivative

**DOI:** 10.1107/S2056989015006854

**Published:** 2015-04-09

**Authors:** Takeshi Oishi, Keisuke Fukaya, Yu Yamaguchi, Tomoya Sugai, Ami Watanabe, Takaaki Sato, Noritaka Chida

**Affiliations:** a School of Medicine, Keio University, Hiyoshi 4-1-1, Kohoku-ku, Yokohama 223-8521, Japan; b Department of Applied Chemistry, Faculty of Science and Technology, Keio University, Hiyoshi 3-14-1, Kohoku-ku, Yokohama 223-8522, Japan

**Keywords:** crystal structure, hydrogen bonds, taxane skeleton, paclitaxel

## Abstract

The title compounds, C_29_H_38_O_8_·0.25C_5_H_12_, (**A**), C_29_H_38_O_8_, (**B**), and C_29_H_36_O_8_, (**C**), are tetra­cyclic benzoates possessing a taxane skeleton with a fused dioxolane ring as the core structure. In the asymmetric unit of (**A**), there are two independent benzoate mol­ecules (A and A′) and a half mol­ecule of solvent pentane disordered about an inversion center. The mol­ecular conformations of (**A**), (**B**) and (**C**) are similar except for the flexible meth­oxy­meth­oxy group. The cyclo­hexane, cyclo­hexene and central cyclo­octane rings adopt chair, half-chair and chair–chair (extended crown) forms, respectively. The dioxolane rings are essentially planar, while the dioxolane ring of A′ is slightly twisted from the mean plane. In the crystal of (**A**), inter­molecular O—H⋯O, C—H⋯O and C—H⋯π inter­actions link the independent benzoates alternately, forming a chain structure. In the crystals of (**B**) and (**C**), mol­ecules are linked through O—H⋯O and C—H⋯π inter­actions, and C—H⋯O hydrogen bonds, respectively, into similar chains. Further, weak inter­molecular C—H⋯O inter­actions connect the chains into a three-dimensional network in (**A**) and a sheet in (**B**), whereas no other interactions are observed for (**C**).

## Abstract 2

In the title three compounds, the ring conformations of tetra­cycles are similar; each tetra­cycle adopts essentially planar, chair, half-chair and chair–chair forms. In the crystals, mol­ecules are linked into similar chains by inter­molecular hydrogen bonds.

## Chemical context   

**Figure 1:**
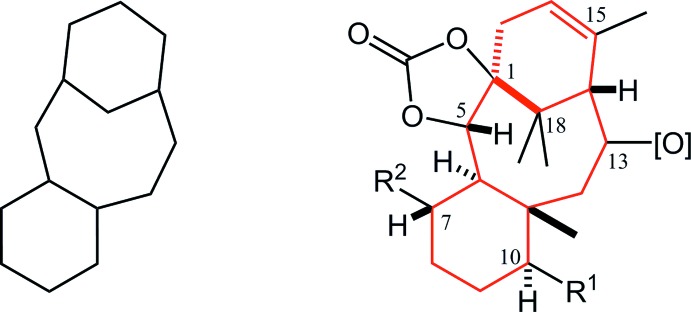
Left: Structure of the tri­cyclo­[9.3.1.0^3,8^]penta­decane (taxane) skeleton; Right: Core structure of the title compounds. Red lines indicate the taxane skeleton. *R* ^1^ = OC(=O)Ph, *R* ^2^ = OCH_2_OCH_3_.

Paclitaxel is a well-known natural diterpenoid containing a taxane framework (tri­cyclo­[9.3.1.0^3,8^]penta­decane; Fig. 1 ▸), with potent anti­tumor activity (Wall & Wani, 1995 ▸). The complicated structure and significant bioactivity have attracted chemical and medicinal inter­est. Recently, we reported the crystal structure of the precursor for cyclization to build the taxane skeleton (Oishi *et al.*, 2015 ▸; §4), which was obtained in a synthetic study of paclitaxel. The cyclization reaction was accomplished (Fukaya *et al.*, 2015 ▸) to afford strained tetra­cyclic benzoates (**A**) and its 13-epimer (**B**), then further oxidation gave a ketone (**C**).
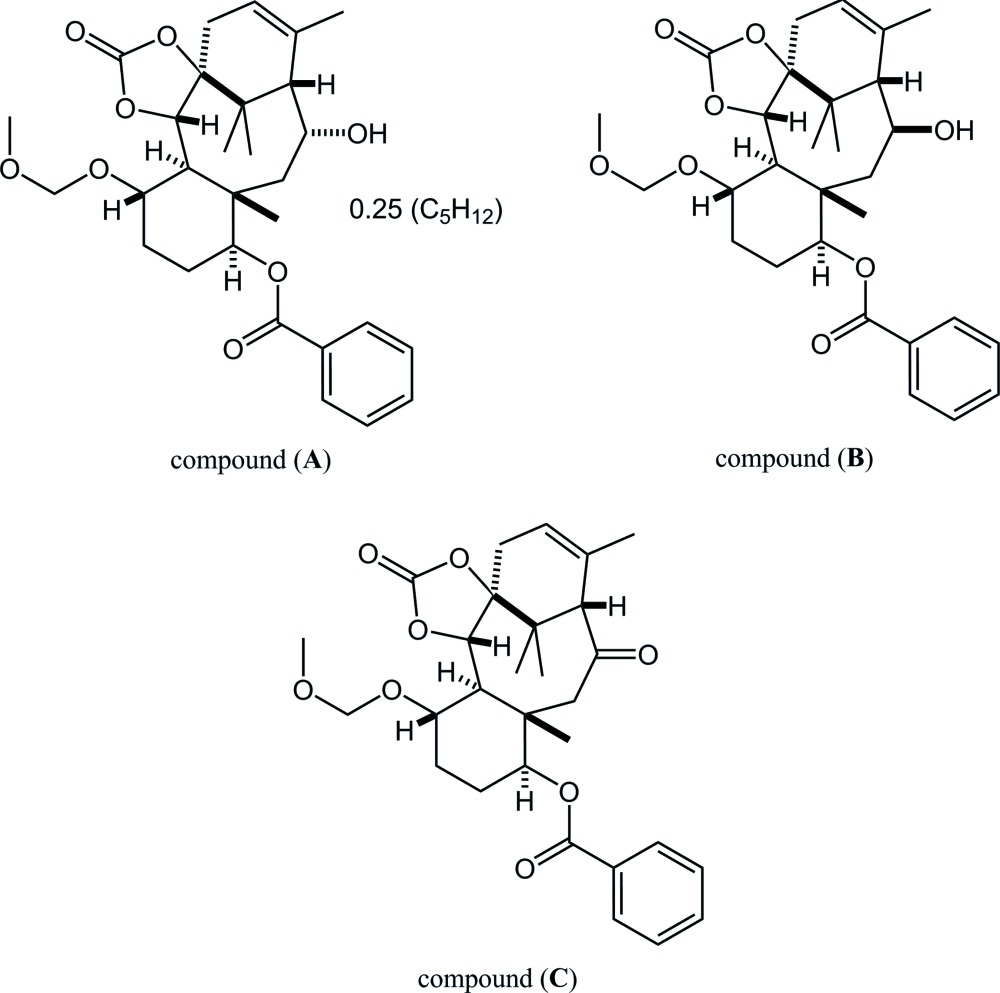


## Structural commentary   

**Figure 2:**
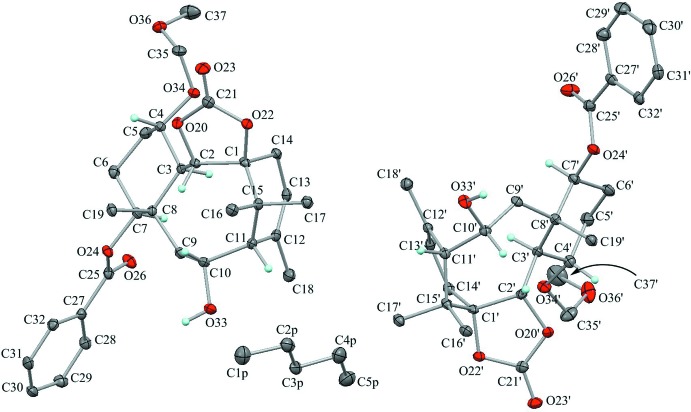
The asymmetric unit of compound (**A**) with the atom labelling. Displacement ellipsoids are drawn at the 30% probability level. The left benzoate mol­ecule has been moved by a symmetry operation of (−*x* + 1, −*y* + 1, −*z* + 1) from its original position. The pentane solvent mol­ecule is disordered by symmetry over two sites with occupancy 0.50. Only H atoms connected to O and chiral C atoms are shown for clarity.

**Figure 5:**
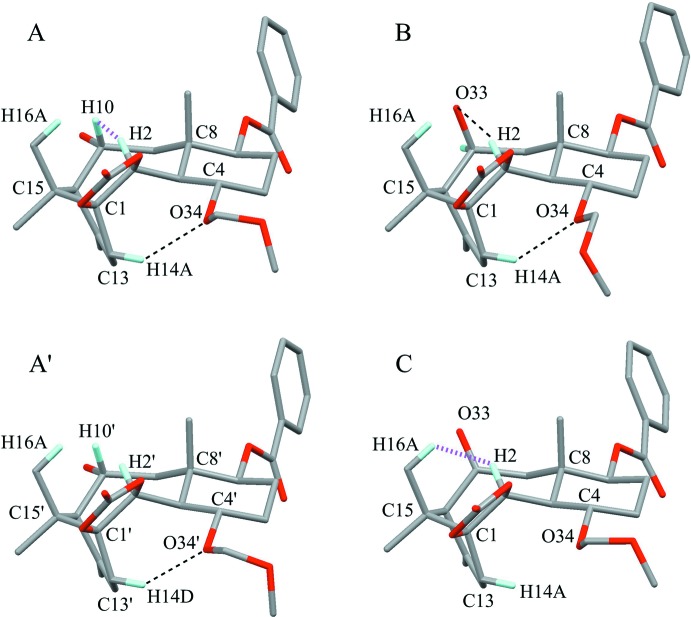
The mol­ecular conformations of compounds, (**A**), (**B**) and (**C**). In (**A**), there are two independent benzoates indicated as A (C1–C37) and A′ (C1′–C37′). Black dashed lines indicate the intra­molecular C—H⋯O inter­actions. Purple dotted lines indicate intra­molecular H⋯H short contacts. For clarity, only H atoms involved in these inter­actions are shown.

The asymmetric units of the title compounds, (**A**), (**B**) and (**C**), are shown in Figs. 2 ▸, 3 ▸ and 4 ▸, respectively. Their mol­ecular conformations are similar except for the flexible meth­oxy­meth­oxy group (Fig. 5 ▸).

### (±)-(1*SR*,5*SR*,6*SR*,7*SR*,10*SR*,11*SR*,13*RS*,14*SR*)-13-Hy­droxy-7-meth­oxy­meth­oxy-3-oxo-11,15,18,18-tetra­methyl-2,4-dioxa­tetra­cyclo­[12.3.1.0^1,5^.0^6,11^]octa­dec-15-en-10-yl benzoate, (**A**)   

The two independent mol­ecules, A (C1–C37) and A′ (C1′–C37′), adopt slightly different conformations. The pentane solvent mol­ecule is disordered around the center of symmetry. The dioxolane ring in A (C1/C2/O20/C21/O22) is essentially planar with a maximum deviation of 0.0434 (18) Å for atom C1, while the dioxolane ring in A′ (C1′/C2′/O20′/C21′/O22′) shows a flattened twisted form with puckering parameters of *Q*(2) = 0.0713 (17) Å and *φ*(2) = 47.8 (14)°. Atoms C1′ and C2′ deviate from the mean plane of the other atoms by 0.104 (4) and −0.019 (4) Å, respectively.

The cyclo­hexane rings (C3–C8 in A and C3′–C8′ in A′) adopt chair forms with puckering parameters of *Q* = 0.5728 (19) Å, *θ* = 174.96 (19)°, *φ* = 352 (2)°, *Q*(2) = 0.0508 (19) Å and *Q*(3) = – 0.5705 (19) Å for the C3–C8 ring, and *Q* = 0.570 (2) Å, *θ* = 6.68 (19)°, *φ* = 181.2 (16)°, *Q*(2) = 0.0691 (19) Å and *Q*(3) = 0.5656 (19) Å for the C3′–C8′ ring. The larger substituents (C3—C2 and C3′—C2′; C4—O34 and C4′—C34′; C7—O24 and C7′—C24′) are in the equatorial positions, but substituents on quaternary carbons (C8—C9 and C8′—C9′) are slightly tilted from the equatorial positions with angles to the *Cremer & Pople plane* of 59.74 (13) and 59.59 (13)°, respectively.

The cyclo­hexene ring in A (C1/C14/C13/C12/C11/C15) adopts a half-chair form with puckering parameters of *Q* = 0.5419 (18) Å, *θ* = 50.2 (2)°, *φ* = 318.9 (3)°, *Q*(2) = 0.4162 (19) Å and *Q*(3) = −0.3470 (19) Å. Atoms C1 and C15 deviate from the mean plane of the other four atoms by 0.272 (3) and −0.547 (3) Å, respectively. The cyclo­hexene ring in A′ (C1′/C14′/C13′/C12′/C11′/C15′) also adopts a half-chair form with puckering parameters of *Q* = 0.5364 (19) Å, *θ* = 129.8 (2)°, *φ* = 138.8 (3)°, *Q*(2) = 0.4124 (19) Å and *Q*(3) = −0.3431 (19) Å. Atoms C1′ and C15′ deviate from the mean plane of other atoms by −0.268 (3) and 0.543 (3) Å, respectively.

The central cyclo­octane ring in A (C1–C3/C8–C11/C15) adopts a chair–chair (an extended crown) form with puckering parameters of *Q* = 0.8995 (18) Å, *Q*(2) = 0.3441 (18) Å, *φ*(2) = 305.1 (3)°, *Q*(3) = 0.0632 (18) Å, *φ*(3) = 180.8 (16)° and *Q*(4) = −0.8286 (18) Å. The cyclo­octane ring in A′ (C1′–C3′/C8′–C11′) also adopts a similar form with puckering parameters of *Q* = 0.8940 (18) Å, *Q*(2) = 0.3432 (18) Å, *φ*(2) = 130.3 (3)°, *Q*(3) = 0.0866 (19) Å, *φ*(3) = 11.1 (12)° and *Q*(4) = 0.8209 (18) Å. There is a short intra­molecular contact of 1.93 Å between atoms H2 and H10 in A, while the distance between H2′ and H10′ in A′ is 2.05 Å. The meth­oxy­meth­oxy groups (O34/C35/O36/C37 in A and O34′/C35′/O36′/C37′ in A′) show helical forms with weak intra­molecular C—H⋯O inter­actions (Fig. 5 ▸, Table 1 ▸).

### (±)-(1*SR*,5*SR*,6*SR*,7*SR*,10*SR*,11*SR*,13*SR*,14*SR*)-13-Hy­droxy-7-meth­oxy­meth­oxy-3-oxo-11,15,18,18-tetra­methyl-2,4-dioxa­tetra­cyclo­[12.3.1.0^1,5^.0^6,11^]octa­dec-15-en-10-yl benzoate, (**B**)   

Compound (**B**) is the C10-epimer of (**A**). The dioxolane ring in (**B**) (C1/C2/O20/C21/O22) is essentially planar with a maximum deviation of 0.0124 (11) Å for atom O22. The cyclo­hexane ring (C3–C8) adopts a chair form with puckering parameters of *Q* = 0.564 (2) Å, *θ* = 4.1 (2)°, *φ* = 124 (3)°, *Q*(2) = 0.039 (2) Å and *Q*(3) = 0.562 (2) Å. The larger substituents (C3—C2, C4—O34 and C7—O24) are in the equatorial positions, while the substituents on quaternary carbon (C8—C9) is slightly tilted from the equatorial position with an angle to the *Cremer & Pople plane* of 57.89 (13)°.

The cyclo­hexene ring (C1/C14/C13/C12/C11/C15) adopts a half-chair form with puckering parameters of *Q* = 0.540 (2) Å, *θ* = 130.1 (2)°, *φ* = 136.3 (3)°, *Q*(2) = 0.413 (2) Å and *Q*(3) = −0.348 (2) Å. Atoms C1 and C15 deviate from the mean plane of the other four atoms by 0.237 (4) and −0.575 (4) Å, respectively. The central cyclo­octane ring (C1–C3/C8–C11/C15) adopts a chair-chair form with puckering parameters of *Q* = 0.863 (2) Å, *Q*(2) = 0.283 (2) Å, *φ*(2) = 126.7 (4)°, *Q*(3) = 0.113 (2) Å, *φ*(3) = 23.1 (10)° and *Q*(4) = 0.807 (2) Å. The elongated bond lengths of 1.584 (3) Å for C10—C11 and 1.571 (3) Å for C11—C15, and the extraordinary *sp* ^3^ angle of 126.80 (17)° for C8—C9—C10 suggest strain in the fused ring system. There are intra­molecular C—H⋯O inter­actions (C2—H2⋯O33 and C14—H14*A*⋯O34; Table 2 ▸).

### (±)-(1*SR*,5*SR*,6*SR*,7*SR*,10*SR*,11*SR*,14*SR*)-3,13-Dioxo-7-meth­oxy­meth­oxy-11,15,18,18-tetra­methyl-2,4-dioxa­tetra­cyclo­[12.3.1.0^1,5^.0^6,11^]octa­dec-15-en-10-yl benzoate, (**C**)   

Compound (**C**) is the C10-oxo derivative of (**A**) and (**B**). The dioxolane ring in (**C**) (C1/C2/O20/C21/O22) is essentially planar with a maximum deviation of 0.0280 (12) Å for atom O22. The cyclo­hexane ring (C3–C8) adopts a chair form with puckering parameters of *Q* = 0.563 (2) Å, *θ* = 5.9 (2)°, *φ* = 227 (2)°, *Q*(2) = 0.056 (2) Å and *Q*(3) = 0.560 (2) Å. The substituents including that on the quaternary carbon (C3—C2, C4—O34, C7—O24 and C8—C9) are in the equatorial positions.

The cyclo­hexene ring (C1/C14/C13/C12/C11/C15) adopts a half-chair form with puckering parameters of *Q* = 0.533 (2) Å, *θ* = 131.7 (2)°, *φ* = 135.3 (3)°, *Q*(2) = 0.398 (2) Å and *Q*(3) = −0.354 (2) Å. Atoms C1 and C15 deviate from the mean plane of the other four atoms by 0.222 (4) and −0.577 (4) Å, respectively. The central cyclo­octane ring (C1–C3/C8–C11/C15) adopts a chair-chair form with puckering parameters of *Q* = 0.898 (2) Å, *Q*(2) = 0.311 (2) Å, *φ*(2) = 113.2 (4)°, *Q*(3) = 0.066 (2) Å, *φ*(3) = 353 (2)° and *Q*(4) = 0.839 (2) Å. There is a short intra­molecular contact of 1.88 Å between the atoms H2 and H16*A*.

## Supra­molecular features   

### Compound (**A**)   

**Figure 6:**
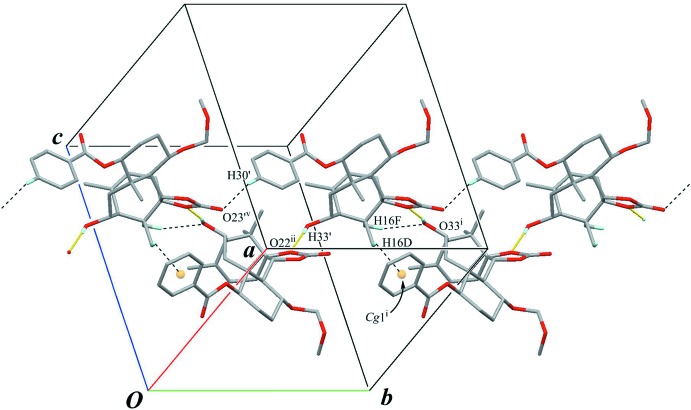
A partial packing view of (**A**) showing the chain structure. Yellow lines indicate the inter­molecular O—H⋯O hydrogen bonds, generating *C*(7) chains. Black dashed lines indicate the weak inter­molecular C—H⋯O and C—H⋯π inter­actions. *Cg*1 is the centroid of the C27–C32 benzene ring. Only H atoms involved in hydrogen bonds are shown for clarity. The pentane solvent mol­ecules have been omitted. [Symmetry codes: (i) −*x* + 1, −*y* + 2, −*z* + 1; (ii) −*x* + 1, −*y* + 1, −*z* + 1; (v) *x*, *y* − 1, *z*.]

The crystal packing is stabilized by inter­molecular O—H⋯O hydrogen bonds (O33—H33⋯O22′^i^ and O33′—H33′⋯O22^ii^; Table 1 ▸) connecting the A and A′ mol­ecules alternately to form a chain with a *C*(7) motif running along the *b* axis (Fig. 6 ▸). Further inter­molecular weak C—H⋯O and C—H⋯π inter­actions (C17′—C17F⋯O33^i^, C30′—H30′⋯O23′^v^ and C16′—H16*D*⋯*Cg*1^i^; Table 1 ▸) support the chain structure. Inter­estingly, the geometric data for the corresponding inter­actions (C17—H17*C*⋯O33′^ii^, C30—H30⋯O23^vi^ and C16—H16*A*⋯*Cg*1′) are 2.76 Å for H17*C*⋯O33′^ii^, 2.80 Å for H30⋯O23^vi^ and 2.95 Å for H16—*Cg*1′, and 118.8° for C30—H30⋯O23^vi^ and 119° for C16—H16*A*⋯*Cg*1′, which are out of the range for proper values of a hydrogen bond [symmetry code: (vi) *x*, *y* + 1, *z*; *Cg*1′ is the centroid of the C27′–C32′ benzene ring.]

**Figure 7:**
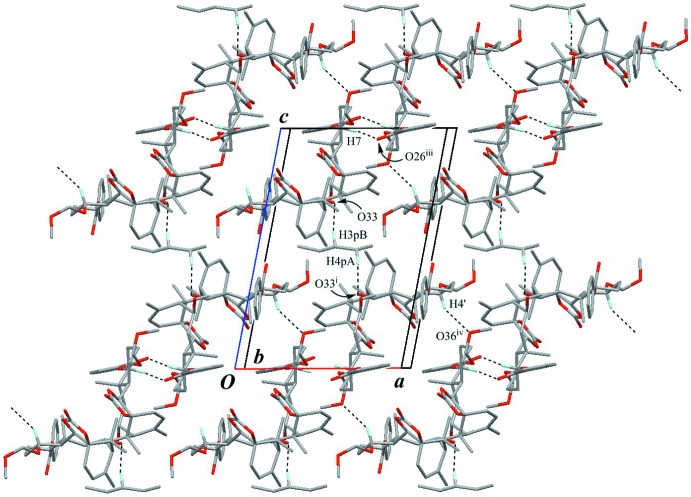
A packing diagram of (**A**) viewed down to *b* axis showing a three-dimensional network. The chains (projected as *butterfly*-like shapes) are connected by the inter­molecular C—H⋯O inter­actions (black dashed lines). Only H atoms involved in hydrogen bonds are shown for clarity. [Symmetry codes: (iii) −*x* + 1, −*y* + 2, −*z* + 2; (iv) *x* + 1, *y*, *z* − 1.]

The chains are inter­locked by a pair of inter­molecular C—H⋯O hydrogen bonds (C7—H7⋯O26^iii^; Table 1 ▸) with an 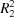
(10) graph-set motif, forming a tape parallel to (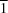
01) and along the *b* axis (Fig. 7 ▸). The adjacent tapes are connected by inter­molecular C—H⋯O inter­actions (C4′—H4′⋯O36^iv^; Table 1 ▸), forming a layer parallel to (001). Among the layers, disordered solvent pentane mol­ecules are held by weak inter­molecular C—H⋯O inter­actions (C3*P*—H3*PB*⋯O33 and C4*P*—H4*PA*⋯O33^i^; Table 1 ▸), constructing a three-dimensional architecture.

### Compound (**B**)   

**Figure 8:**
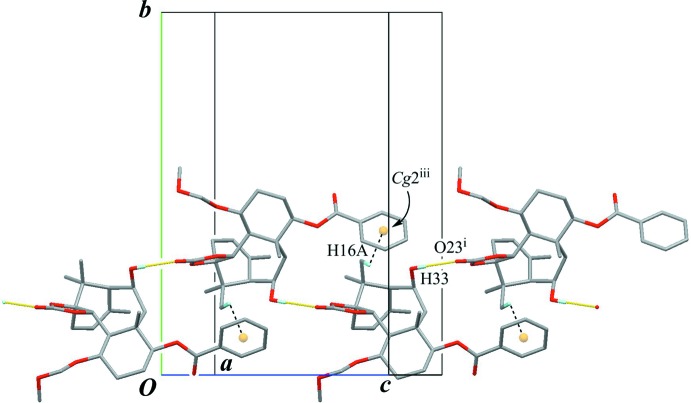
A partial packing view of (**B**) showing a chain structure. The inter­molecular O—H⋯O hydrogen bonds (yellow lines) link the enanti­omers alternately, generating *C*(9) chains. In the chain, further inter­molecular weak C—H⋯π inter­actions (black dashed lines) are also observed. *Cg*2 is the centroid of the C27–C32 phenyl ring. Only H atoms involved in hydrogen bonds are shown for clarity. [Symmetry codes: (i) *x* + 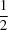, −*y* + 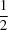, −*z* + 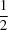; (iii) *x* − 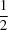, −*y* + 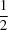, *z* − 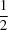.]

**Figure 9:**
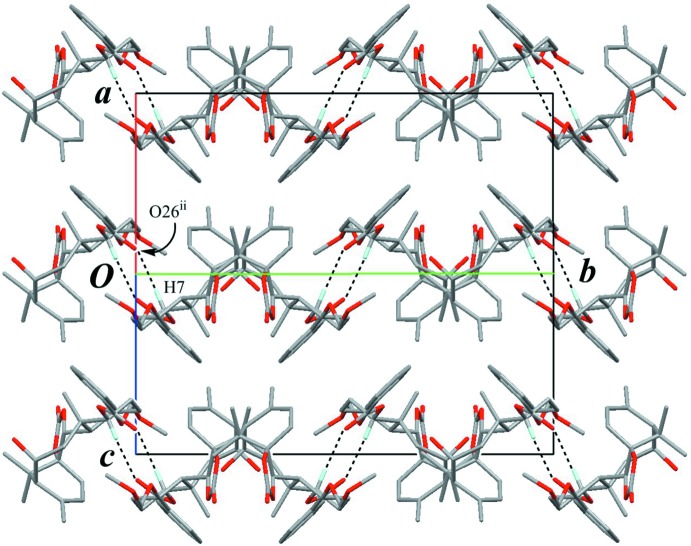
A packing diagram of (**B**) viewed along [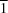
0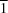], showing parallel sheets. The chains (projected as *fly*-like shapes) are connected by pairs of inter­molecular C—H⋯O inter­actions (black dashed lines), forming sheets parallel to (10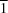). Only H atoms involved in hydrogen bonds are shown for clarity. [Symmetry codes: (ii) −*x* + 2, −*y*, −*z* + 2.]

The crystal packing is stabilized by an inter­molecular O—H⋯O hydrogen bond (O33—H33⋯O23^i^; Table 2 ▸) connecting the enanti­omers alternately to form a chain with a *C*(9) motif along [101] (Fig. 8 ▸). Further, an inter­molecular weak C—H⋯π inter­action (C16—H16*A*⋯*Cg*2^iii^; Table 2 ▸) supports the chain formation. The chains are connected by a pair of inter­molecular C—H⋯O hydrogen bonds (C7—H7⋯O26^ii^; Fig. 9 ▸, Table 2 ▸) with an 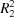
(10) graph-set motif, forming a sheet parallel to (10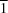).

### Compound (**C**)   

**Figure 10:**
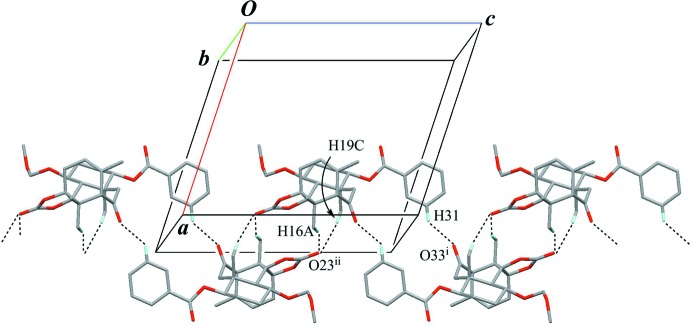
A partial packing view of (**C**) showing the chain structure. Inter­molecular C—H⋯O inter­actions (blacked dashed lines) link the enanti­omers. Only H atoms involved in hydrogen bonds are shown for clarity. [Symmetry codes: (i) −*x* + 2, −*y* + 1, −*z* + 2; (ii) −*x* + 2, −*y* + 1, −*z* + 1.]

The crystal packing is stabilized by a pair of inter­molecular C—H⋯O inter­actions (C31—H31⋯O33^i^; Table 3 ▸) with an 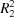
(22) graph-set motif, forming an inversion dimer (Figs. 10 ▸ and 11 ▸). The dimers are further linked into a chain along the *c* axis by inter­molecular C—H⋯O inter­actions (C19—H19*C*⋯O23^ii^ and C16—H16*A*⋯O23^ii^; Table 3 ▸) with 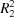
(16) and 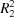
(14) graph-set motifs, respectively. There is an inter­molecular O36⋯C25^iii^ short contact of 3.012 (3) Å involving the carbonyl group of the benzoyl moiety [symmetry code: (iii) *x*, −*y* + 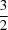, *z* − 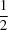].

## Database survey   

**Figure 12:**
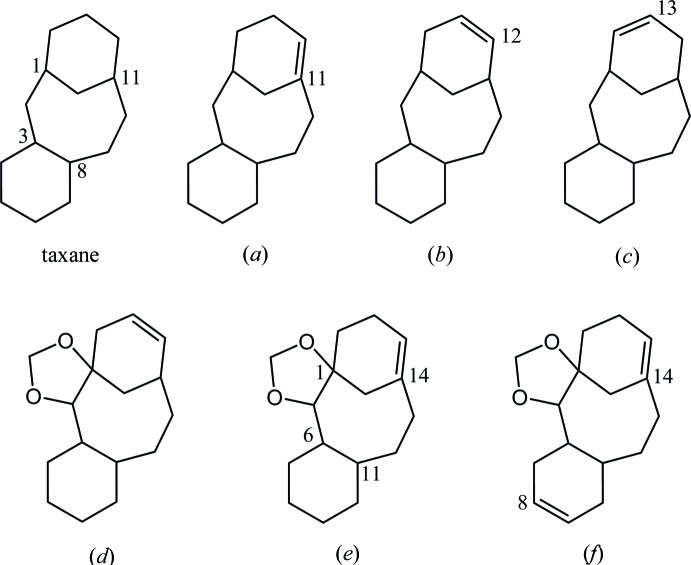
Core structures for database survey; tri­cyclo­[9.3.1.0^3,8^]penta­decane (taxane) and its (*a*) 11-ene, (*b*) 12-ene and (*c*) 13-ene derivatives, (*d*) tetra­cyclic core of the title compounds and (*e*) its regioisomer of olefin and (*f*) de­hydro derivative of regioisomer.

In the Cambridge Structural Database (CSD, Version 5.36, November 2014; Groom & Allen, 2014 ▸), 85 structures containing a tri­cyclo­[9.3.1.0^3,8^]penta­dec-11-ene skeleton, (*a*), are found (Fig. 12 ▸). These include a large number of paclitaxels and its analogues, and one compound (NEGBOQ; Poujol *et al.*, 1997 ▸) containing a 2,4-dioxa­tetra­cyclo[12.3.1.0^1,5^.0^6,11^]octa­dec-14-ene skeleton, (*e*), which is an olefin regioisomer for the tetra­cyclic core of the title compound, (*d*). On the other hand, there are two related structures (PAHTEZ; Mendoza *et al.*, 2011 ▸, and RIYTAW; Wilde *et al.*, 2014 ▸) containing a tri­cyclo­[9.3.1.0^3,8^]penta­dec-12-ene skeleton, (*b*), and one related structure (SOJWOD; Paquette & Zhao, 1998 ▸) for a tri­cyclo­[9.3.1.0^3,8^]penta­dec-13-ene skeleton, (*c*).

Another tetra­cyclic taxoid (ILIQUP; Ohba *et al.*, 2003 ▸), which was unexpectedly generated by a cyclization reaction in our previous study, is closely related to the title compound. Additionally, a precursor of cyclization obtained in our previous study is also available (NOTROF; Oishi *et al.*, 2015 ▸). Another compound, closely related to the title compounds with a 2,4-dioxa­tetra­cyclo­[12.3.1.0^1,5^.0^6,11^]octa­deca-8,14-diene skeleton, (*f*), was reported in the literature (Nicolaou *et al.*, 1995 ▸), but was not deposited in the CSD.

## Synthesis and crystallization   

The title compounds were obtained in a synthetic study on paclitaxel (Fukaya *et al.*, 2015 ▸). The cyclo­hexene unit (C1/C14/C13/C12/C11/C15) was synthesized according to a reported procedure (Nicolaou *et al.*, 1995 ▸), and coupled with the substituted cyclo­hexane unit (C3–C8) prepared from 3-methyl­anisole by a Shapiro reaction (Nicolaou *et al.*, 1995 ▸). Further manipulation of the functional groups and cyclization reaction afforded the tetra­cyclic benzoates (**A**) and its C10-epimer (**B**), which were oxidized into ketone (**C**). Each compound was purified by silica gel chromatography. Colorless crystals of (**A**) were grown from a benzene solution under a pentane-saturated atmosphere by slow evaporation at ambient temperature. Similarly, colorless crystals of (**B**) and (**C**) were obtained in the same manner.

## Refinement   

Crystal data, data collection and structure refinement details are summarized in Table 4 ▸. C-bound H atoms were positioned geometrically with C—H = 0.95–1.00 Å, and constrained to ride on their parent atoms with *U*_iso_(H) = 1.2*U*_eq_(C) or 1.5*U*_eq_(methyl C). The H atom of hy­droxy group was placed guided by difference maps, with O—H = 0.84 Å and with *U*_iso_(H) = 1.5*U*_eq_(O).

## Supplementary Material

Crystal structure: contains datablock(s) global, A, B, C. DOI: 10.1107/S2056989015006854/is5395sup1.cif

Structure factors: contains datablock(s) A. DOI: 10.1107/S2056989015006854/is5395Asup2.hkl

Structure factors: contains datablock(s) B. DOI: 10.1107/S2056989015006854/is5395Bsup3.hkl

Structure factors: contains datablock(s) C. DOI: 10.1107/S2056989015006854/is5395Csup4.hkl

CCDC references: 1057985, 1057984, 1057983

Additional supporting information:  crystallographic information; 3D view; checkCIF report

## Figures and Tables

**Figure 3:**
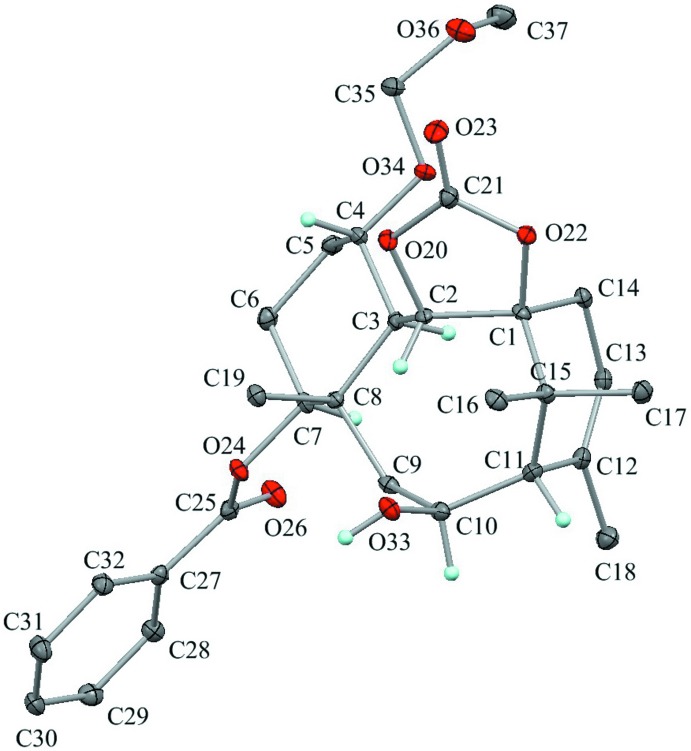
The asymmetric unit of compound (**B**) with the atom labelling. Displacement ellipsoids are drawn at the 30% probability level. Only H atoms connected to O and chiral C atoms are shown for clarity.

**Figure 4:**
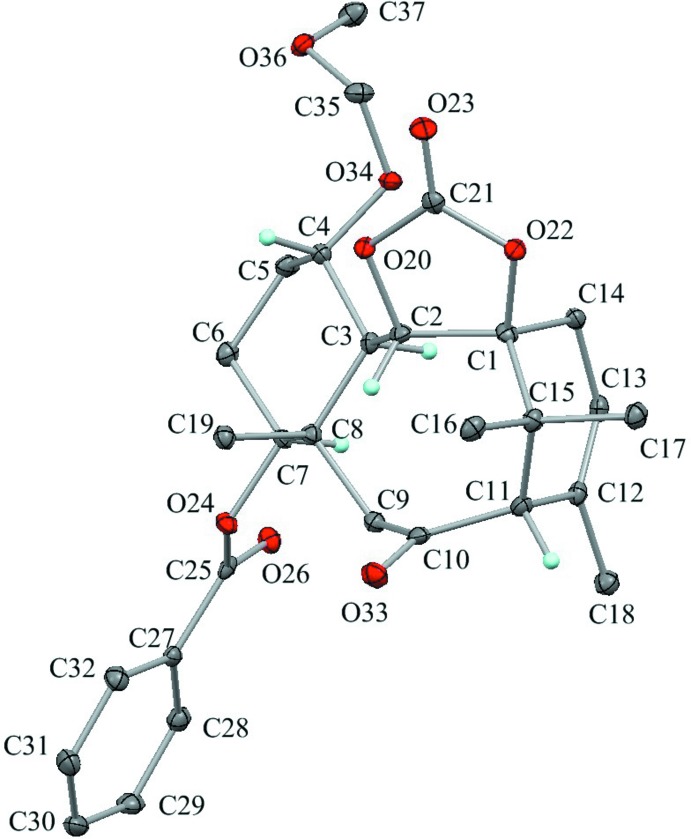
The asymmetric unit of compound (**C**) with the atom labelling. Displacement ellipsoids are drawn at the 30% probability level. Only H atoms connected to O and chiral C atoms are shown for clarity.

**Figure 11:**
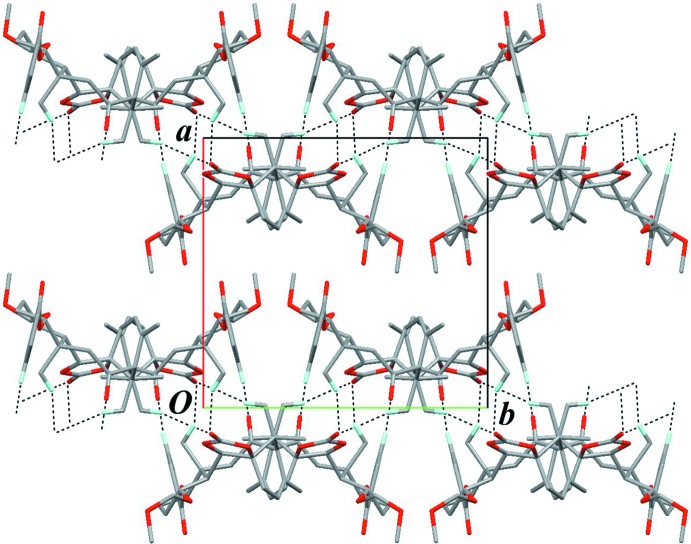
A packing diagram of (**C**) viewed down the *c* axis. Black dashed lines indicate the inter­molecular C—H⋯O inter­actions. Overlapped mol­ecules (projection as a *spider*-like shape) do not constitute the same chain. A half body of the *spider* is only linked to the adjacent inverted one.

**Table 1:** Hydrogen-bond geometry (Å, °) for **A** [Chem scheme1] *Cg*1 is the centroid of the C27–C32 benzene ring.

*D*—H⋯*A*	*D*—H	H⋯*A*	*D*⋯*A*	*D*—H⋯*A*
C14—H14*A*⋯O34	0.99	2.47	3.320 (2)	143
C14′—H14*D*⋯O34′	0.99	2.36	3.221 (2)	145
O33—H33⋯O22′^i^	0.84	2.05	2.8563 (17)	160
O33′—H33′⋯O22^ii^	0.84	2.05	2.8839 (16)	169
C7—H7⋯O26^iii^	1.00	2.28	3.236 (2)	159
C4′—H4′⋯O36^iv^	1.00	2.40	3.311 (2)	151
C17′—H17*F*⋯O33^i^	0.98	2.48	3.431 (2)	164
C30′—H30′⋯O23′^v^	0.95	2.53	3.453 (2)	163
C3*P*—H3*PB*⋯O33	0.99	2.49	3.369 (9)	149
C4*P*—H4*PA*⋯O33^i^	0.99	2.41	3.291 (12)	148
C16′—H16*D*⋯*Cg*1^i^	0.98	2.85	3.5315 (19)	127

Symmetry codes: (i) 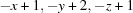; (ii) 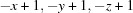; (iii) 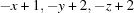; (iv) 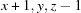; (v) 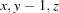.

**Table 2:** Hydrogen-bond geometry (Å, °) for **B** [Chem scheme1] *Cg*2 is the centroid of the C27–C32 benzene ring.

*D*—H⋯*A*	*D*—H	H⋯*A*	*D*⋯*A*	*D*—H⋯*A*
C2—H2⋯O33	0.98	2.27	3.200 (2)	157
C14—H14*A*⋯O34	0.97	2.47	3.293 (2)	142
O33—H33⋯O23^i^	0.82	1.96	2.7823 (19)	179
C7—H7⋯O26^ii^	0.98	2.50	3.353 (2)	145
C16—H16*A*⋯*Cg*2^iii^	0.98	2.93	3.594 (2)	128

Symmetry codes: (i) 
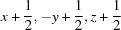
; (ii) 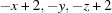; (iii) 
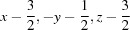
.

**Table 3:** Hydrogen-bond geometry (Å, °) for **C** [Chem scheme1]

*D*—H⋯*A*	*D*—H	H⋯*A*	*D*⋯*A*	*D*—H⋯*A*
C31—H31⋯O33^i^	0.95	2.35	3.147 (3)	141
C19—H19*C*⋯O23^ii^	0.98	2.43	3.310 (3)	149
C16—H16*A*⋯O23^ii^	0.98	2.56	3.491 (3)	158

Symmetry codes: (i) 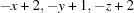; (ii) 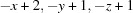.

**Table 4:** Experimental details

	**A**	**B**	**C**
Crystal data			
Chemical formula	C_29_H_38_O_8_·0.25C_5_H_12_	C_29_H_38_O_8_	C_29_H_36_O_8_
*M*_r_	532.64	514.59	512.59
Crystal system, space group	Triclinic, *P* 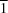	Monoclinic, *P*2_1_/*n*	Monoclinic, *P*2_1_/*c*
Temperature (K)	90	90	90
*a*, *b*, *c* (Å)	11.3343 (5), 15.4666 (7), 16.4870 (8)	9.3612 (6), 19.6336 (15), 14.1965 (9)	13.2416 (8), 13.1779 (8), 15.2428 (8)
α, β, γ (°)	85.1124 (14), 78.3773 (14), 78.5231 (15)	90, 101.762 (2), 90	90, 109.387 (2), 90
*V* (Å^3^)	2771.3 (2)	2554.4 (3)	2509.0 (3)
*Z*	4	4	4
Radiation type	Mo *K*α	Mo *K*α	Mo *K*α
μ (mm^−1^)	0.09	0.10	0.10
Crystal size (mm)	0.32 × 0.21 × 0.17	0.23 × 0.23 × 0.14	0.22 × 0.14 × 0.09

Data collection			
Diffractometer	Bruker D8 Venture	Bruker D8 Venture	Bruker D8 Venture
Absorption correction	Multi-scan (*SADABS*; Bruker, 2014 ▸)	Multi-scan (*SADABS*; Bruker, 2014 ▸)	Multi-scan (*SADABS*; Bruker, 2014 ▸)
*T*_min_, *T*_max_	0.97, 0.98	0.98, 0.99	0.92, 0.99
No. of measured, independent and observed [*I* > 2σ(*I*)] reflections	50997, 9735, 7339	23252, 4480, 3212	22512, 4395, 3050
*R*_int_	0.041	0.058	0.063
(sin θ/λ)_max_ (Å^−1^)	0.595	0.595	0.595

Refinement			
*R*[*F* ^2^ > 2σ(*F* ^2^)], *wR*(*F* ^2^), *S*	0.040, 0.099, 1.02	0.043, 0.111, 1.02	0.044, 0.105, 0.96
No. of reflections	9735	4480	4395
No. of parameters	726	340	339
H-atom treatment	H-atom parameters constrained	H-atom parameters constrained	H-atom parameters constrained
Δρ_max_, Δρ_min_ (e Å^−3^)	0.27, −0.24	0.51, −0.21	0.29, −0.25

Computer programs: *APEX2* and *SAINT* (Bruker, 2014 ▸), *SHELXS2013* (Sheldrick, 2008 ▸), *SHELXL2014* (Sheldrick, 2015 ▸), *Mercury* (Macrae *et al.*, 2006 ▸), *publCIF* (Westrip, 2010 ▸) and *PLATON* (Spek, 2009 ▸).

## supplementary crystallographic information

### (A) (±)-(1SR,5SR,6SR,7SR,10SR,11SR,13RS,14SR)-13-Hydroxy-7-methoxymethoxy-11,15,18,18-tetramethyl-3-oxo-2,4-dioxatetracyclo[12.3.1.01,5.06,11]octadec-15-en-10-yl benzoate pentane 0.25-solvate Crystal data

**Table:**

C_29_H_38_O_8_·0.25C_5_H_12_	*F*(000) = 1146
*M**_r_* = 532.64	*D*_x_ = 1.277 Mg m^−^^3^
Triclinic, *P*1	Melting point: 509.2 K
*a* = 11.3343 (5) Å	Mo *K*α radiation, λ = 0.71073 Å
*b* = 15.4666 (7) Å	Cell parameters from 9428 reflections
*c* = 16.4870 (8) Å	θ = 2.4–25.0°
α = 85.1124 (14)°	µ = 0.09 mm^−^^1^
β = 78.3773 (14)°	*T* = 90 K
γ = 78.5231 (15)°	Prism, colorless
*V* = 2771.3 (2) Å^3^	0.32 × 0.21 × 0.17 mm
*Z* = 4	

### (A) (±)-(1SR,5SR,6SR,7SR,10SR,11SR,13RS,14SR)-13-Hydroxy-7-methoxymethoxy-11,15,18,18-tetramethyl-3-oxo-2,4-dioxatetracyclo[12.3.1.01,5.06,11]octadec-15-en-10-yl benzoate pentane 0.25-solvate Data collection

**Table:**

Bruker D8 Venture diffractometer	9735 independent reflections
Radiation source: fine-focus sealed tube	7339 reflections with *I* > 2σ(*I*)
Multilayered confocal mirror monochromator	*R*_int_ = 0.041
Detector resolution: 8.333 pixels mm^-1^	θ_max_ = 25.0°, θ_min_ = 2.2°
φ and ω scans	*h* = −13→13
Absorption correction: multi-scan (*SADABS*; Bruker, 2014)	*k* = −18→18
*T*_min_ = 0.97, *T*_max_ = 0.98	*l* = −19→19
50997 measured reflections	

### (A) (±)-(1SR,5SR,6SR,7SR,10SR,11SR,13RS,14SR)-13-Hydroxy-7-methoxymethoxy-11,15,18,18-tetramethyl-3-oxo-2,4-dioxatetracyclo[12.3.1.01,5.06,11]octadec-15-en-10-yl benzoate pentane 0.25-solvate Refinement

**Table:**

Refinement on *F*^2^	Primary atom site location: structure-invariant direct methods
Least-squares matrix: full	Secondary atom site location: difference Fourier map
*R*[*F*^2^ > 2σ(*F*^2^)] = 0.040	Hydrogen site location: inferred from neighbouring sites
*wR*(*F*^2^) = 0.099	H-atom parameters constrained
*S* = 1.02	*w* = 1/[σ^2^(*F*_o_^2^) + (0.039*P*)^2^ + 1.4256*P*] where *P* = (*F*_o_^2^ + 2*F*_c_^2^)/3
9735 reflections	(Δ/σ)_max_ = 0.008
726 parameters	Δρ_max_ = 0.27 e Å^−^^3^
0 restraints	Δρ_min_ = −0.24 e Å^−^^3^

### (A) (±)-(1SR,5SR,6SR,7SR,10SR,11SR,13RS,14SR)-13-Hydroxy-7-methoxymethoxy-11,15,18,18-tetramethyl-3-oxo-2,4-dioxatetracyclo[12.3.1.01,5.06,11]octadec-15-en-10-yl benzoate pentane 0.25-solvate Special details

**Table:**

Experimental. *M.p.* 507.2–509.2 K (not corrected); IR (film) 3502, 2950, 1799, 1717, 1451, 1272, 1098, 1055, 713 cm^-1^; ^1^H NMR (500 MHz, CDCl_3_) *δ* (p.p.m.) 8.03–8.00 (m, 2H), 7.60–7.55 (m, 1H), 7.48–7.43 (m, 2H), 5.50 (bs, 1H), 4.78 (d, *J* = 7.2 Hz, 1H), 4.71 (dd, *J* = 11.3, 4.3 Hz, 1H), 4.58 (d, *J* = 7.2 Hz, 1H), 4.50 (d, *J* = 3.7 Hz, 1H), 4.31 (dd, *J* = 9.0, 2.0 Hz, 1H), 3.56 (ddd, *J* = 10.7, 10.7, 5.2 Hz, 1H), 3.35 (s, 3H), 2.87 (bd, *J* = 18.3 Hz, 1H), 2.41–2.30 (m, 2H), 2.11 (s, 1H), 2.08 (dd, *J* = 10.7, 3.7 Hz, 1H), 1.89 (dddd, *J* = 12.7, 4.3, 4.0, 4.0 Hz, 1H), 1.77 (s, 3H), 1.72–1.50 (m, 4H), 1.29 (s, 3H), 1.25 (s, 3H), 1.17 (s, 3H); ^13^C NMR (125 MHz, CDCl_3_) *δ* (p.p.m.) 165.9 (C), 154.1 (C), 136.7 (C), 133.4 (CH), 130.3 (C), 129.7 (CH), 128.6 (CH), 120.9 (CH), 97.8 (CH_2_), 87.1 (C), 80.0 (CH), 78.1 (CH), 75.0 (CH), 67.1 (CH), 60.6 (CH), 55.9 (CH_3_), 47.8 (CH_2_), 46.2 (CH), 42.6 (C), 40.2 (C), 31.9 (CH_2_), 31.2 (CH_2_), 25.9 (CH_3_), 25.1 (CH_3_), 24.7 (CH_2_), 19.8 (CH_3_), 13.6 (CH_3_); LRMS (EI) *m/z* 514 (*M*^+^, 4%), 483 (1), 469 (1), 453 (1), 409 (4), 393 (12), 348 (1), 332 (1), 121 (83), 105 (100), 77 (67); HRMS (EI) *m/z* calcd for C_29_H_38_O_8_^+^ [*M*]^+^ 514.2567, found 514.2545.
Geometry. All e.s.d.'s (except the e.s.d. in the dihedral angle between two l.s. planes) are estimated using the full covariance matrix. The cell e.s.d.'s are taken into account individually in the estimation of e.s.d.'s in distances, angles and torsion angles; correlations between e.s.d.'s in cell parameters are only used when they are defined by crystal symmetry. An approximate (isotropic) treatment of cell e.s.d.'s is used for estimating e.s.d.'s involving l.s. planes.
Refinement. Refinement of *F*^2^ against ALL reflections. The weighted *R*-factor *wR* and goodness of fit *S* are based on *F*^2^, conventional *R*-factors *R* are based on *F*, with *F* set to zero for negative *F*^2^. The threshold expression of *F*^2^ > σ(*F*^2^) is used only for calculating *R*-factors(gt) *etc*. and is not relevant to the choice of reflections for refinement. *R*-factors based on *F*^2^ are statistically about twice as large as those based on *F*, and *R*- factors based on ALL data will be even larger.Problematic three reflections with |*I*(obs)-*I*(calc)|/σW(*I*) greater than 10 (1 1 0, 9 9 11 and 5 3 11) have been omitted in the final refinement.

### (A) (±)-(1SR,5SR,6SR,7SR,10SR,11SR,13RS,14SR)-13-Hydroxy-7-methoxymethoxy-11,15,18,18-tetramethyl-3-oxo-2,4-dioxatetracyclo[12.3.1.01,5.06,11]octadec-15-en-10-yl benzoate pentane 0.25-solvate Fractional atomic coordinates and isotropic or equivalent isotropic displacement parameters (Å^2^)

**Table:**

	*x*	*y*	*z*	*U*_iso_*/*U*_eq_	Occ. (<1)
C1	0.39192 (15)	0.71883 (11)	0.80546 (10)	0.0182 (4)	
C2	0.29323 (15)	0.76771 (11)	0.87419 (10)	0.0178 (4)	
H2	0.2366	0.8123	0.8455	0.021*	
C3	0.33237 (15)	0.81694 (11)	0.93856 (10)	0.0167 (4)	
H3	0.4227	0.8146	0.9212	0.02*	
C4	0.31165 (16)	0.77206 (11)	1.02534 (11)	0.0199 (4)	
H4	0.2234	0.767	1.0428	0.024*	
C5	0.34803 (17)	0.82311 (11)	1.08803 (11)	0.0228 (4)	
H5A	0.4373	0.8225	1.0739	0.027*	
H5B	0.3297	0.7943	1.1439	0.027*	
C6	0.27850 (17)	0.91845 (11)	1.08866 (11)	0.0231 (4)	
H6A	0.1893	0.9196	1.1049	0.028*	
H6B	0.3035	0.9513	1.1293	0.028*	
C7	0.30713 (16)	0.96120 (11)	1.00303 (10)	0.0192 (4)	
H7	0.3973	0.9604	0.9885	0.023*	
C8	0.27009 (15)	0.91625 (11)	0.93435 (10)	0.0169 (4)	
C9	0.32337 (16)	0.96195 (11)	0.85154 (10)	0.0196 (4)	
H9B	0.285	1.0253	0.8542	0.024*	
H9A	0.4116	0.9585	0.8511	0.024*	
C10	0.31409 (16)	0.93271 (11)	0.76616 (10)	0.0196 (4)	
H10	0.2326	0.9153	0.7703	0.024*	
C11	0.41687 (15)	0.85809 (11)	0.72360 (10)	0.0184 (4)	
H11	0.4345	0.8795	0.6642	0.022*	
C12	0.53785 (16)	0.85012 (11)	0.75254 (10)	0.0201 (4)	
C13	0.58295 (16)	0.77927 (11)	0.79574 (11)	0.0211 (4)	
H13	0.659	0.7782	0.812	0.025*	
C14	0.52260 (15)	0.70092 (11)	0.82067 (11)	0.0201 (4)	
H14A	0.522	0.6854	0.8802	0.024*	
H14B	0.5706	0.6499	0.7888	0.024*	
C15	0.38464 (15)	0.76528 (11)	0.72002 (10)	0.0191 (4)	
C16	0.25946 (16)	0.77064 (12)	0.69574 (11)	0.0225 (4)	
H16A	0.1941	0.7916	0.7423	0.034*	
H16B	0.2515	0.712	0.6819	0.034*	
H16C	0.2529	0.8118	0.6476	0.034*	
C17	0.47951 (17)	0.71477 (12)	0.65165 (11)	0.0237 (4)	
H17B	0.4675	0.742	0.5973	0.036*	
H17C	0.4692	0.6531	0.6547	0.036*	
H17A	0.5623	0.7171	0.6594	0.036*	
C18	0.60684 (17)	0.92447 (12)	0.72667 (12)	0.0278 (4)	
H18A	0.6397	0.9233	0.667	0.042*	
H18B	0.6745	0.9177	0.7569	0.042*	
H18C	0.5513	0.9809	0.7392	0.042*	
C19	0.13055 (15)	0.92911 (11)	0.94717 (11)	0.0214 (4)	
H19C	0.1076	0.9005	0.9034	0.032*	
H19A	0.0958	0.9924	0.945	0.032*	
H19B	0.0988	0.9027	1.0013	0.032*	
O20	0.22400 (11)	0.70057 (8)	0.91336 (7)	0.0214 (3)	
C21	0.26240 (16)	0.62580 (12)	0.87360 (11)	0.0216 (4)	
O22	0.35432 (11)	0.63298 (7)	0.80929 (7)	0.0215 (3)	
O23	0.22106 (12)	0.56013 (8)	0.89208 (8)	0.0298 (3)	
O24	0.24272 (10)	1.05356 (7)	1.00196 (7)	0.0201 (3)	
C25	0.30152 (16)	1.11360 (11)	1.02153 (10)	0.0197 (4)	
O26	0.39773 (11)	1.09511 (8)	1.04546 (8)	0.0283 (3)	
C27	0.23598 (16)	1.20589 (11)	1.01040 (10)	0.0194 (4)	
C28	0.29728 (17)	1.27379 (12)	1.01677 (11)	0.0231 (4)	
H28	0.3782	1.2602	1.0278	0.028*	
C29	0.24120 (18)	1.36072 (12)	1.00719 (11)	0.0272 (4)	
H29	0.2837	1.4068	1.0113	0.033*	
C30	0.12343 (18)	1.38076 (12)	0.99168 (11)	0.0275 (4)	
H30	0.0847	1.4407	0.9855	0.033*	
C31	0.06173 (17)	1.31401 (12)	0.98513 (12)	0.0276 (4)	
H31	−0.0195	1.3281	0.9748	0.033*	
C32	0.11788 (16)	1.22643 (12)	0.99364 (11)	0.0226 (4)	
H32	0.0758	1.1806	0.988	0.027*	
O33	0.32219 (12)	1.00634 (8)	0.70805 (8)	0.0276 (3)	
H33	0.2664	1.049	0.7246	0.041*	
O34	0.38701 (11)	0.68492 (7)	1.01813 (7)	0.0218 (3)	
C35	0.33439 (17)	0.61646 (12)	1.06299 (11)	0.0248 (4)	
H35A	0.2477	0.625	1.0568	0.03*	
H35B	0.3774	0.5597	1.0387	0.03*	
O36	0.33867 (12)	0.61094 (8)	1.14683 (7)	0.0288 (3)	
C37	0.4588 (2)	0.58010 (15)	1.16272 (15)	0.0452 (6)	
H37B	0.5132	0.6193	1.1335	0.068*	
H37C	0.489	0.5201	1.1432	0.068*	
H37A	0.457	0.5795	1.2224	0.068*	
C1'	0.80525 (16)	0.74195 (11)	0.31038 (11)	0.0190 (4)	
C2'	0.90913 (15)	0.67289 (11)	0.26359 (10)	0.0185 (4)	
H2'	0.8711	0.6423	0.2274	0.022*	
C3'	0.98195 (15)	0.60049 (11)	0.31378 (10)	0.0182 (4)	
H3'	0.9397	0.6052	0.3731	0.022*	
C4'	1.11310 (16)	0.61437 (11)	0.30982 (11)	0.0225 (4)	
H4'	1.1551	0.6192	0.2506	0.027*	
C5'	1.18605 (17)	0.53955 (12)	0.35504 (12)	0.0274 (4)	
H5'A	1.1505	0.5397	0.415	0.033*	
H5'B	1.2718	0.5483	0.3478	0.033*	
C6'	1.18424 (17)	0.45122 (12)	0.32163 (12)	0.0265 (4)	
H6'A	1.2218	0.4501	0.262	0.032*	
H6'B	1.2321	0.4028	0.3514	0.032*	
C7'	1.05332 (16)	0.43877 (11)	0.33368 (11)	0.0216 (4)	
H7'	1.0172	0.4405	0.3942	0.026*	
C8'	0.97153 (15)	0.50882 (11)	0.28678 (10)	0.0188 (4)	
C9'	0.83902 (16)	0.49154 (11)	0.31629 (11)	0.0205 (4)	
H9'A	0.8245	0.4886	0.3776	0.025*	
H9'B	0.84	0.4314	0.2995	0.025*	
C10'	0.72291 (15)	0.55088 (11)	0.29171 (11)	0.0193 (4)	
H10'	0.7401	0.5667	0.2309	0.023*	
C11'	0.66133 (16)	0.63631 (11)	0.33978 (10)	0.0189 (4)	
H11'	0.5715	0.6377	0.3454	0.023*	
C12'	0.68111 (15)	0.62936 (11)	0.42879 (11)	0.0191 (4)	
C13'	0.74589 (16)	0.68057 (11)	0.45440 (11)	0.0211 (4)	
H13'	0.7548	0.6732	0.5108	0.025*	
C14'	0.80599 (16)	0.74896 (12)	0.40147 (11)	0.0220 (4)	
H14D	0.8917	0.7418	0.4094	0.026*	
H14E	0.7625	0.8084	0.4191	0.026*	
C15'	0.67950 (16)	0.72807 (11)	0.29790 (11)	0.0199 (4)	
C16'	0.66754 (17)	0.73683 (12)	0.20653 (11)	0.0237 (4)	
H16D	0.7362	0.6974	0.1743	0.036*	
H16E	0.6686	0.798	0.1857	0.036*	
H16F	0.5902	0.7207	0.2012	0.036*	
C17'	0.57506 (16)	0.79806 (11)	0.34144 (12)	0.0237 (4)	
H17D	0.5714	0.7911	0.4015	0.036*	
H17E	0.497	0.7904	0.3288	0.036*	
H17F	0.5903	0.8572	0.3218	0.036*	
C18'	0.61846 (17)	0.56509 (12)	0.48620 (11)	0.0264 (4)	
H18D	0.5294	0.5838	0.4921	0.04*	
H18E	0.6417	0.5631	0.5406	0.04*	
H18F	0.6433	0.5063	0.4635	0.04*	
C19'	1.01302 (17)	0.49893 (12)	0.19299 (11)	0.0237 (4)	
H19D	1.0253	0.4366	0.1806	0.035*	
H19E	1.0902	0.5205	0.1742	0.035*	
H19F	0.9501	0.5335	0.1642	0.035*	
O20'	0.98878 (11)	0.72516 (7)	0.20899 (7)	0.0218 (3)	
C21'	0.93856 (16)	0.81059 (12)	0.21184 (11)	0.0219 (4)	
O22'	0.83303 (11)	0.82463 (7)	0.26720 (7)	0.0230 (3)	
O23'	0.98135 (12)	0.86793 (8)	0.17053 (8)	0.0291 (3)	
O24'	1.05092 (11)	0.35259 (7)	0.30544 (7)	0.0232 (3)	
C25'	1.05285 (16)	0.28473 (12)	0.36162 (11)	0.0230 (4)	
O26'	1.05309 (14)	0.29155 (9)	0.43359 (8)	0.0366 (4)	
C27'	1.05285 (16)	0.20029 (11)	0.32452 (11)	0.0226 (4)	
C28'	1.03407 (19)	0.12820 (13)	0.37777 (13)	0.0328 (5)	
H28'	1.0236	0.1332	0.4359	0.039*	
C29'	1.0305 (2)	0.04866 (13)	0.34638 (14)	0.0403 (5)	
H29'	1.0167	−0.0006	0.3831	0.048*	
C30'	1.04693 (18)	0.04084 (13)	0.26221 (13)	0.0343 (5)	
H30'	1.044	−0.0137	0.2409	0.041*	
C31'	1.06757 (16)	0.11189 (12)	0.20896 (12)	0.0270 (4)	
H31'	1.0804	0.1059	0.1508	0.032*	
C32'	1.06975 (15)	0.19203 (12)	0.23947 (11)	0.0231 (4)	
H32'	1.0828	0.2412	0.2024	0.028*	
O33'	0.62847 (11)	0.49828 (8)	0.30885 (8)	0.0254 (3)	
H33'	0.6435	0.4595	0.2736	0.038*	
O34'	1.09963 (11)	0.69699 (8)	0.34778 (8)	0.0255 (3)	
C35'	1.1978 (2)	0.74151 (14)	0.32338 (13)	0.0370 (5)	
H35D	1.2264	0.7375	0.2628	0.044*	
H35F	1.1685	0.8047	0.335	0.044*	
O36'	1.29654 (12)	0.70912 (10)	0.36255 (10)	0.0446 (4)	
C37'	1.2687 (2)	0.72296 (17)	0.44817 (15)	0.0529 (7)	
H37D	1.3415	0.6998	0.4725	0.079*	
H37E	1.2021	0.6923	0.4747	0.079*	
H37F	1.2432	0.7864	0.4569	0.079*	
C1P	0.2437 (5)	0.9991 (4)	0.5096 (4)	0.0466 (14)	0.5
H1PA	0.2192	1.0095	0.569	0.07*	0.5
H1PB	0.1943	0.9599	0.4945	0.07*	0.5
H1PC	0.2307	1.0555	0.4777	0.07*	0.5
C2P	0.3778 (6)	0.9566 (6)	0.4905 (6)	0.0345 (17)	0.5
H2PA	0.4002	0.942	0.4314	0.041*	0.5
H2PB	0.3906	0.9007	0.5242	0.041*	0.5
C3P	0.4624 (5)	1.0156 (5)	0.5077 (5)	0.036 (3)	0.5
H3PA	0.4536	1.0696	0.471	0.043*	0.5
H3PB	0.4357	1.0338	0.5656	0.043*	0.5
C4P	0.5962 (7)	0.9719 (8)	0.4947 (7)	0.044 (2)	0.5
H4PA	0.6228	0.9528	0.437	0.053*	0.5
H4PB	0.6055	0.9184	0.5322	0.053*	0.5
C5P	0.6788 (7)	1.0321 (6)	0.5108 (6)	0.047 (2)	0.5
H5PA	0.6741	1.0834	0.4715	0.07*	0.5
H5PB	0.7636	0.9998	0.5038	0.07*	0.5
H5PC	0.6521	1.0522	0.5675	0.07*	0.5

### (A) (±)-(1SR,5SR,6SR,7SR,10SR,11SR,13RS,14SR)-13-Hydroxy-7-methoxymethoxy-11,15,18,18-tetramethyl-3-oxo-2,4-dioxatetracyclo[12.3.1.01,5.06,11]octadec-15-en-10-yl benzoate pentane 0.25-solvate Atomic displacement parameters (Å^2^)

**Table:**

	*U*^11^	*U*^22^	*U*^33^	*U*^12^	*U*^13^	*U*^23^
C1	0.0196 (9)	0.0124 (9)	0.0231 (9)	−0.0029 (7)	−0.0042 (7)	−0.0035 (7)
C2	0.0196 (9)	0.0145 (9)	0.0193 (9)	−0.0040 (7)	−0.0026 (7)	−0.0005 (7)
C3	0.0138 (9)	0.0148 (9)	0.0206 (9)	−0.0020 (7)	−0.0009 (7)	−0.0039 (7)
C4	0.0203 (10)	0.0151 (9)	0.0226 (10)	−0.0024 (7)	−0.0008 (8)	−0.0021 (7)
C5	0.0287 (11)	0.0216 (10)	0.0192 (9)	−0.0069 (8)	−0.0045 (8)	−0.0011 (8)
C6	0.0247 (10)	0.0228 (10)	0.0223 (10)	−0.0053 (8)	−0.0021 (8)	−0.0077 (8)
C7	0.0174 (9)	0.0146 (9)	0.0248 (10)	−0.0008 (7)	−0.0030 (8)	−0.0042 (7)
C8	0.0149 (9)	0.0166 (9)	0.0186 (9)	−0.0031 (7)	−0.0010 (7)	−0.0029 (7)
C9	0.0190 (9)	0.0154 (9)	0.0241 (10)	−0.0014 (7)	−0.0038 (8)	−0.0037 (7)
C10	0.0195 (9)	0.0159 (9)	0.0219 (9)	−0.0012 (7)	−0.0034 (7)	0.0019 (7)
C11	0.0189 (9)	0.0188 (9)	0.0163 (9)	−0.0027 (7)	−0.0013 (7)	−0.0010 (7)
C12	0.0188 (9)	0.0220 (10)	0.0183 (9)	−0.0031 (7)	0.0001 (7)	−0.0049 (8)
C13	0.0153 (9)	0.0255 (10)	0.0219 (9)	−0.0019 (7)	−0.0030 (7)	−0.0048 (8)
C14	0.0200 (10)	0.0172 (9)	0.0211 (9)	0.0022 (7)	−0.0035 (8)	−0.0035 (7)
C15	0.0185 (9)	0.0194 (9)	0.0188 (9)	−0.0027 (7)	−0.0025 (7)	−0.0033 (7)
C16	0.0255 (10)	0.0225 (10)	0.0209 (9)	−0.0042 (8)	−0.0070 (8)	−0.0026 (8)
C17	0.0263 (10)	0.0220 (10)	0.0220 (10)	−0.0020 (8)	−0.0038 (8)	−0.0044 (8)
C18	0.0232 (10)	0.0267 (11)	0.0327 (11)	−0.0050 (8)	−0.0034 (8)	−0.0006 (9)
C19	0.0178 (9)	0.0188 (9)	0.0269 (10)	−0.0013 (7)	−0.0025 (8)	−0.0071 (8)
O20	0.0205 (7)	0.0192 (7)	0.0249 (7)	−0.0077 (5)	0.0003 (5)	−0.0052 (5)
C21	0.0230 (10)	0.0206 (10)	0.0225 (10)	−0.0041 (8)	−0.0071 (8)	−0.0023 (8)
O22	0.0245 (7)	0.0153 (6)	0.0241 (7)	−0.0046 (5)	−0.0015 (6)	−0.0038 (5)
O23	0.0360 (8)	0.0211 (7)	0.0353 (8)	−0.0141 (6)	−0.0050 (6)	−0.0013 (6)
O24	0.0193 (6)	0.0147 (6)	0.0271 (7)	−0.0027 (5)	−0.0042 (5)	−0.0065 (5)
C25	0.0187 (10)	0.0205 (10)	0.0204 (9)	−0.0044 (8)	−0.0021 (8)	−0.0051 (7)
O26	0.0252 (8)	0.0221 (7)	0.0403 (8)	−0.0016 (6)	−0.0127 (6)	−0.0069 (6)
C27	0.0233 (10)	0.0180 (9)	0.0158 (9)	−0.0026 (7)	−0.0012 (7)	−0.0033 (7)
C28	0.0240 (10)	0.0229 (10)	0.0236 (10)	−0.0040 (8)	−0.0059 (8)	−0.0054 (8)
C29	0.0372 (12)	0.0194 (10)	0.0271 (10)	−0.0077 (8)	−0.0070 (9)	−0.0044 (8)
C30	0.0347 (12)	0.0173 (10)	0.0254 (10)	0.0029 (8)	−0.0011 (9)	−0.0009 (8)
C31	0.0184 (10)	0.0287 (11)	0.0314 (11)	0.0018 (8)	−0.0019 (8)	0.0013 (9)
C32	0.0212 (10)	0.0233 (10)	0.0233 (10)	−0.0071 (8)	−0.0011 (8)	−0.0018 (8)
O33	0.0342 (8)	0.0182 (7)	0.0259 (7)	0.0014 (6)	−0.0026 (6)	0.0019 (6)
O34	0.0255 (7)	0.0151 (6)	0.0237 (7)	−0.0036 (5)	−0.0030 (5)	0.0006 (5)
C35	0.0298 (11)	0.0187 (10)	0.0274 (10)	−0.0073 (8)	−0.0076 (8)	0.0029 (8)
O36	0.0321 (8)	0.0298 (7)	0.0229 (7)	−0.0051 (6)	−0.0048 (6)	0.0051 (6)
C37	0.0452 (14)	0.0441 (14)	0.0534 (15)	−0.0124 (11)	−0.0287 (12)	0.0146 (11)
C1'	0.0211 (10)	0.0112 (9)	0.0243 (10)	−0.0038 (7)	−0.0033 (8)	0.0000 (7)
C2'	0.0175 (9)	0.0180 (9)	0.0209 (9)	−0.0068 (7)	−0.0034 (7)	0.0008 (7)
C3'	0.0185 (9)	0.0171 (9)	0.0181 (9)	−0.0020 (7)	−0.0031 (7)	−0.0003 (7)
C4'	0.0204 (10)	0.0221 (10)	0.0261 (10)	−0.0044 (8)	−0.0056 (8)	−0.0031 (8)
C5'	0.0193 (10)	0.0299 (11)	0.0341 (11)	−0.0042 (8)	−0.0097 (8)	0.0017 (9)
C6'	0.0237 (10)	0.0219 (10)	0.0318 (11)	0.0030 (8)	−0.0085 (8)	0.0011 (8)
C7'	0.0242 (10)	0.0176 (9)	0.0224 (10)	−0.0009 (7)	−0.0048 (8)	−0.0031 (7)
C8'	0.0189 (9)	0.0175 (9)	0.0199 (9)	−0.0019 (7)	−0.0043 (7)	−0.0022 (7)
C9'	0.0240 (10)	0.0144 (9)	0.0238 (10)	−0.0033 (7)	−0.0053 (8)	−0.0034 (7)
C10'	0.0207 (10)	0.0168 (9)	0.0221 (9)	−0.0063 (7)	−0.0045 (8)	−0.0030 (7)
C11'	0.0170 (9)	0.0172 (9)	0.0231 (9)	−0.0039 (7)	−0.0032 (7)	−0.0038 (7)
C12'	0.0179 (9)	0.0163 (9)	0.0214 (9)	−0.0003 (7)	−0.0010 (7)	−0.0039 (7)
C13'	0.0213 (10)	0.0232 (10)	0.0181 (9)	−0.0021 (8)	−0.0031 (8)	−0.0043 (8)
C14'	0.0216 (10)	0.0195 (10)	0.0260 (10)	−0.0042 (7)	−0.0041 (8)	−0.0067 (8)
C15'	0.0202 (10)	0.0153 (9)	0.0245 (10)	−0.0023 (7)	−0.0047 (8)	−0.0042 (7)
C16'	0.0235 (10)	0.0203 (10)	0.0271 (10)	−0.0014 (8)	−0.0074 (8)	0.0002 (8)
C17'	0.0211 (10)	0.0181 (10)	0.0319 (11)	−0.0022 (7)	−0.0052 (8)	−0.0039 (8)
C18'	0.0265 (11)	0.0252 (10)	0.0263 (10)	−0.0050 (8)	−0.0014 (8)	−0.0033 (8)
C19'	0.0258 (10)	0.0194 (10)	0.0243 (10)	−0.0001 (8)	−0.0046 (8)	−0.0029 (8)
O20'	0.0205 (7)	0.0184 (7)	0.0248 (7)	−0.0039 (5)	−0.0021 (5)	0.0033 (5)
C21'	0.0204 (10)	0.0216 (10)	0.0260 (10)	−0.0064 (8)	−0.0079 (8)	0.0007 (8)
O22'	0.0226 (7)	0.0148 (6)	0.0303 (7)	−0.0043 (5)	−0.0021 (6)	0.0009 (5)
O23'	0.0298 (8)	0.0242 (7)	0.0345 (8)	−0.0115 (6)	−0.0059 (6)	0.0067 (6)
O24'	0.0294 (7)	0.0154 (6)	0.0235 (7)	0.0007 (5)	−0.0069 (5)	−0.0016 (5)
C25'	0.0204 (10)	0.0235 (10)	0.0255 (11)	−0.0023 (8)	−0.0077 (8)	0.0011 (8)
O26'	0.0626 (10)	0.0264 (8)	0.0268 (8)	−0.0135 (7)	−0.0178 (7)	0.0013 (6)
C27'	0.0184 (10)	0.0195 (10)	0.0301 (11)	−0.0006 (7)	−0.0075 (8)	−0.0021 (8)
C28'	0.0420 (13)	0.0270 (11)	0.0291 (11)	−0.0067 (9)	−0.0059 (9)	−0.0012 (9)
C29'	0.0523 (14)	0.0217 (11)	0.0457 (14)	−0.0112 (10)	−0.0027 (11)	0.0000 (10)
C30'	0.0337 (12)	0.0260 (11)	0.0440 (13)	−0.0073 (9)	−0.0034 (10)	−0.0121 (10)
C31'	0.0191 (10)	0.0304 (11)	0.0315 (11)	−0.0022 (8)	−0.0030 (8)	−0.0115 (9)
C32'	0.0141 (9)	0.0242 (10)	0.0305 (11)	−0.0008 (7)	−0.0048 (8)	−0.0029 (8)
O33'	0.0259 (7)	0.0194 (7)	0.0340 (8)	−0.0078 (5)	−0.0057 (6)	−0.0095 (6)
O34'	0.0230 (7)	0.0230 (7)	0.0334 (7)	−0.0082 (5)	−0.0084 (6)	−0.0017 (6)
C35'	0.0419 (13)	0.0394 (13)	0.0365 (12)	−0.0245 (10)	−0.0065 (10)	−0.0009 (10)
O36'	0.0221 (8)	0.0521 (10)	0.0644 (11)	−0.0103 (7)	−0.0081 (7)	−0.0220 (8)
C37'	0.0500 (15)	0.0592 (16)	0.0602 (17)	−0.0112 (12)	−0.0312 (13)	−0.0112 (13)
C1P	0.040 (3)	0.054 (4)	0.045 (3)	−0.006 (3)	−0.007 (3)	−0.007 (3)
C2P	0.047 (5)	0.029 (3)	0.026 (3)	−0.002 (4)	−0.006 (4)	−0.005 (2)
C3P	0.057 (9)	0.022 (5)	0.021 (3)	0.000 (6)	0.008 (6)	−0.008 (3)
C4P	0.054 (7)	0.042 (4)	0.031 (3)	−0.003 (5)	0.002 (4)	−0.004 (3)
C5P	0.047 (6)	0.051 (5)	0.040 (3)	−0.011 (5)	−0.003 (5)	0.008 (3)

### (A) (±)-(1SR,5SR,6SR,7SR,10SR,11SR,13RS,14SR)-13-Hydroxy-7-methoxymethoxy-11,15,18,18-tetramethyl-3-oxo-2,4-dioxatetracyclo[12.3.1.01,5.06,11]octadec-15-en-10-yl benzoate pentane 0.25-solvate Geometric parameters (Å, º)

**Table:**

C1—O22	1.466 (2)	C3'—C8'	1.555 (2)
C1—C14	1.519 (2)	C3'—H3'	1.0
C1—C15	1.536 (2)	C4'—O34'	1.438 (2)
C1—C2	1.551 (2)	C4'—C5'	1.516 (2)
C2—O20	1.455 (2)	C4'—H4'	1.0
C2—C3	1.538 (2)	C5'—C6'	1.521 (3)
C2—H2	1.0	C5'—H5'A	0.99
C3—C4	1.531 (2)	C5'—H5'B	0.99
C3—C8	1.560 (2)	C6'—C7'	1.506 (3)
C3—H3	1.0	C6'—H6'A	0.99
C4—O34	1.445 (2)	C6'—H6'B	0.99
C4—C5	1.517 (2)	C7'—O24'	1.456 (2)
C4—H4	1.0	C7'—C8'	1.542 (2)
C5—C6	1.527 (2)	C7'—H7'	1.0
C5—H5A	0.99	C8'—C19'	1.534 (2)
C5—H5B	0.99	C8'—C9'	1.552 (2)
C6—C7	1.509 (2)	C9'—C10'	1.552 (2)
C6—H6A	0.99	C9'—H9'A	0.99
C6—H6B	0.99	C9'—H9'B	0.99
C7—O24	1.470 (2)	C10'—O33'	1.440 (2)
C7—C8	1.544 (2)	C10'—C11'	1.565 (2)
C7—H7	1.0	C10'—H10'	1.0
C8—C19	1.527 (2)	C11'—C12'	1.520 (2)
C8—C9	1.550 (2)	C11'—C15'	1.557 (2)
C9—C10	1.544 (2)	C11'—H11'	1.0
C9—H9B	0.99	C12'—C13'	1.326 (2)
C9—H9A	0.99	C12'—C18'	1.496 (2)
C10—O33	1.429 (2)	C13'—C14'	1.495 (2)
C10—C11	1.567 (2)	C13'—H13'	0.95
C10—H10	1.0	C14'—H14D	0.99
C11—C12	1.520 (2)	C14'—H14E	0.99
C11—C15	1.559 (2)	C15'—C16'	1.532 (2)
C11—H11	1.0	C15'—C17'	1.539 (2)
C12—C13	1.327 (2)	C16'—H16D	0.98
C12—C18	1.502 (2)	C16'—H16E	0.98
C13—C14	1.496 (2)	C16'—H16F	0.98
C13—H13	0.95	C17'—H17D	0.98
C14—H14A	0.99	C17'—H17E	0.98
C14—H14B	0.99	C17'—H17F	0.98
C15—C16	1.535 (2)	C18'—H18D	0.98
C15—C17	1.538 (2)	C18'—H18E	0.98
C16—H16A	0.98	C18'—H18F	0.98
C16—H16B	0.98	C19'—H19D	0.98
C16—H16C	0.98	C19'—H19E	0.98
C17—H17B	0.98	C19'—H19F	0.98
C17—H17C	0.98	O20'—C21'	1.331 (2)
C17—H17A	0.98	C21'—O23'	1.192 (2)
C18—H18A	0.98	C21'—O22'	1.342 (2)
C18—H18B	0.98	O24'—C25'	1.339 (2)
C18—H18C	0.98	C25'—O26'	1.201 (2)
C19—H19C	0.98	C25'—C27'	1.489 (3)
C19—H19A	0.98	C27'—C28'	1.383 (3)
C19—H19B	0.98	C27'—C32'	1.390 (3)
O20—C21	1.330 (2)	C28'—C29'	1.385 (3)
C21—O23	1.193 (2)	C28'—H28'	0.95
C21—O22	1.343 (2)	C29'—C30'	1.375 (3)
O24—C25	1.340 (2)	C29'—H29'	0.95
C25—O26	1.207 (2)	C30'—C31'	1.374 (3)
C25—C27	1.487 (2)	C30'—H30'	0.95
C27—C32	1.391 (2)	C31'—C32'	1.383 (3)
C27—C28	1.391 (2)	C31'—H31'	0.95
C28—C29	1.379 (3)	C32'—H32'	0.95
C28—H28	0.95	O33'—H33'	0.84
C29—C30	1.379 (3)	O34'—C35'	1.397 (2)
C29—H29	0.95	C35'—O36'	1.390 (3)
C30—C31	1.380 (3)	C35'—H35D	0.99
C30—H30	0.95	C35'—H35F	0.99
C31—C32	1.386 (3)	O36'—C37'	1.408 (3)
C31—H31	0.95	C37'—H37D	0.98
C32—H32	0.95	C37'—H37E	0.98
O33—H33	0.84	C37'—H37F	0.98
O34—C35	1.401 (2)	C1P—C2P	1.512 (7)
C35—O36	1.388 (2)	C1P—H1PA	0.98
C35—H35A	0.99	C1P—H1PB	0.98
C35—H35B	0.99	C1P—H1PC	0.98
O36—C37	1.419 (2)	C2P—C3P	1.527 (12)
C37—H37B	0.98	C2P—H2PA	0.99
C37—H37C	0.98	C2P—H2PB	0.99
C37—H37A	0.98	C3P—C4P	1.513 (9)
C1'—O22'	1.468 (2)	C3P—H3PA	0.99
C1'—C14'	1.517 (2)	C3P—H3PB	0.99
C1'—C15'	1.539 (2)	C4P—C5P	1.519 (13)
C1'—C2'	1.548 (2)	C4P—H4PA	0.99
C2'—O20'	1.455 (2)	C4P—H4PB	0.99
C2'—C3'	1.539 (2)	C5P—H5PA	0.98
C2'—H2'	1.0	C5P—H5PB	0.98
C3'—C4'	1.531 (2)	C5P—H5PC	0.98
			
O22—C1—C14	106.92 (13)	C4'—C3'—H3'	106.8
O22—C1—C15	109.83 (13)	C2'—C3'—H3'	106.8
C14—C1—C15	110.95 (14)	C8'—C3'—H3'	106.8
O22—C1—C2	101.66 (13)	O34'—C4'—C5'	111.30 (14)
C14—C1—C2	115.83 (14)	O34'—C4'—C3'	105.04 (13)
C15—C1—C2	111.06 (13)	C5'—C4'—C3'	111.67 (15)
O20—C2—C3	111.66 (13)	O34'—C4'—H4'	109.6
O20—C2—C1	104.22 (12)	C5'—C4'—H4'	109.6
C3—C2—C1	119.74 (14)	C3'—C4'—H4'	109.6
O20—C2—H2	106.8	C4'—C5'—C6'	110.44 (15)
C3—C2—H2	106.8	C4'—C5'—H5'A	109.6
C1—C2—H2	106.8	C6'—C5'—H5'A	109.6
C4—C3—C2	112.38 (14)	C4'—C5'—H5'B	109.6
C4—C3—C8	114.30 (13)	C6'—C5'—H5'B	109.6
C2—C3—C8	109.19 (13)	H5'A—C5'—H5'B	108.1
C4—C3—H3	106.8	C7'—C6'—C5'	108.79 (15)
C2—C3—H3	106.8	C7'—C6'—H6'A	109.9
C8—C3—H3	106.8	C5'—C6'—H6'A	109.9
O34—C4—C5	110.55 (14)	C7'—C6'—H6'B	109.9
O34—C4—C3	105.85 (13)	C5'—C6'—H6'B	109.9
C5—C4—C3	111.33 (14)	H6'A—C6'—H6'B	108.3
O34—C4—H4	109.7	O24'—C7'—C6'	109.24 (14)
C5—C4—H4	109.7	O24'—C7'—C8'	107.71 (13)
C3—C4—H4	109.7	C6'—C7'—C8'	114.27 (15)
C4—C5—C6	110.58 (14)	O24'—C7'—H7'	108.5
C4—C5—H5A	109.5	C6'—C7'—H7'	108.5
C6—C5—H5A	109.5	C8'—C7'—H7'	108.5
C4—C5—H5B	109.5	C19'—C8'—C7'	109.93 (14)
C6—C5—H5B	109.5	C19'—C8'—C9'	111.03 (14)
H5A—C5—H5B	108.1	C7'—C8'—C9'	105.96 (14)
C7—C6—C5	108.84 (14)	C19'—C8'—C3'	112.37 (14)
C7—C6—H6A	109.9	C7'—C8'—C3'	106.79 (13)
C5—C6—H6A	109.9	C9'—C8'—C3'	110.48 (14)
C7—C6—H6B	109.9	C8'—C9'—C10'	124.69 (14)
C5—C6—H6B	109.9	C8'—C9'—H9'A	106.2
H6A—C6—H6B	108.3	C10'—C9'—H9'A	106.2
O24—C7—C6	110.10 (13)	C8'—C9'—H9'B	106.2
O24—C7—C8	106.75 (13)	C10'—C9'—H9'B	106.2
C6—C7—C8	114.54 (14)	H9'A—C9'—H9'B	106.3
O24—C7—H7	108.4	O33'—C10'—C9'	106.31 (13)
C6—C7—H7	108.4	O33'—C10'—C11'	102.98 (13)
C8—C7—H7	108.4	C9'—C10'—C11'	119.59 (14)
C19—C8—C7	109.82 (14)	O33'—C10'—H10'	109.1
C19—C8—C9	111.08 (14)	C9'—C10'—H10'	109.1
C7—C8—C9	105.73 (13)	C11'—C10'—H10'	109.1
C19—C8—C3	112.58 (14)	C12'—C11'—C15'	110.97 (14)
C7—C8—C3	106.92 (13)	C12'—C11'—C10'	112.90 (14)
C9—C8—C3	110.40 (13)	C15'—C11'—C10'	119.20 (14)
C10—C9—C8	122.76 (14)	C12'—C11'—H11'	104.0
C10—C9—H9B	106.6	C15'—C11'—H11'	104.0
C8—C9—H9B	106.6	C10'—C11'—H11'	104.0
C10—C9—H9A	106.6	C13'—C12'—C18'	122.25 (16)
C8—C9—H9A	106.6	C13'—C12'—C11'	121.39 (16)
H9B—C9—H9A	106.6	C18'—C12'—C11'	116.29 (15)
O33—C10—C9	108.12 (14)	C12'—C13'—C14'	125.10 (16)
O33—C10—C11	103.00 (13)	C12'—C13'—H13'	117.4
C9—C10—C11	118.56 (14)	C14'—C13'—H13'	117.4
O33—C10—H10	108.9	C13'—C14'—C1'	111.79 (14)
C9—C10—H10	108.9	C13'—C14'—H14D	109.3
C11—C10—H10	108.9	C1'—C14'—H14D	109.3
C12—C11—C15	110.87 (14)	C13'—C14'—H14E	109.3
C12—C11—C10	113.25 (14)	C1'—C14'—H14E	109.3
C15—C11—C10	118.18 (14)	H14D—C14'—H14E	107.9
C12—C11—H11	104.3	C16'—C15'—C1'	112.57 (14)
C15—C11—H11	104.3	C16'—C15'—C17'	105.41 (14)
C10—C11—H11	104.3	C1'—C15'—C17'	111.34 (14)
C13—C12—C18	121.45 (16)	C16'—C15'—C11'	113.16 (14)
C13—C12—C11	121.67 (16)	C1'—C15'—C11'	106.73 (13)
C18—C12—C11	116.82 (15)	C17'—C15'—C11'	107.59 (14)
C12—C13—C14	124.68 (16)	C15'—C16'—H16D	109.5
C12—C13—H13	117.7	C15'—C16'—H16E	109.5
C14—C13—H13	117.7	H16D—C16'—H16E	109.5
C13—C14—C1	111.73 (14)	C15'—C16'—H16F	109.5
C13—C14—H14A	109.3	H16D—C16'—H16F	109.5
C1—C14—H14A	109.3	H16E—C16'—H16F	109.5
C13—C14—H14B	109.3	C15'—C17'—H17D	109.5
C1—C14—H14B	109.3	C15'—C17'—H17E	109.5
H14A—C14—H14B	107.9	H17D—C17'—H17E	109.5
C16—C15—C1	112.74 (14)	C15'—C17'—H17F	109.5
C16—C15—C17	105.90 (14)	H17D—C17'—H17F	109.5
C1—C15—C17	110.91 (14)	H17E—C17'—H17F	109.5
C16—C15—C11	112.53 (14)	C12'—C18'—H18D	109.5
C1—C15—C11	106.34 (13)	C12'—C18'—H18E	109.5
C17—C15—C11	108.40 (14)	H18D—C18'—H18E	109.5
C15—C16—H16A	109.5	C12'—C18'—H18F	109.5
C15—C16—H16B	109.5	H18D—C18'—H18F	109.5
H16A—C16—H16B	109.5	H18E—C18'—H18F	109.5
C15—C16—H16C	109.5	C8'—C19'—H19D	109.5
H16A—C16—H16C	109.5	C8'—C19'—H19E	109.5
H16B—C16—H16C	109.5	H19D—C19'—H19E	109.5
C15—C17—H17B	109.5	C8'—C19'—H19F	109.5
C15—C17—H17C	109.5	H19D—C19'—H19F	109.5
H17B—C17—H17C	109.5	H19E—C19'—H19F	109.5
C15—C17—H17A	109.5	C21'—O20'—C2'	110.28 (13)
H17B—C17—H17A	109.5	O23'—C21'—O20'	124.57 (17)
H17C—C17—H17A	109.5	O23'—C21'—O22'	123.78 (16)
C12—C18—H18A	109.5	O20'—C21'—O22'	111.64 (15)
C12—C18—H18B	109.5	C21'—O22'—C1'	111.54 (13)
H18A—C18—H18B	109.5	C25'—O24'—C7'	117.38 (13)
C12—C18—H18C	109.5	O26'—C25'—O24'	123.87 (17)
H18A—C18—H18C	109.5	O26'—C25'—C27'	124.10 (17)
H18B—C18—H18C	109.5	O24'—C25'—C27'	112.03 (15)
C8—C19—H19C	109.5	C28'—C27'—C32'	119.59 (17)
C8—C19—H19A	109.5	C28'—C27'—C25'	117.84 (17)
H19C—C19—H19A	109.5	C32'—C27'—C25'	122.57 (16)
C8—C19—H19B	109.5	C27'—C28'—C29'	120.05 (19)
H19C—C19—H19B	109.5	C27'—C28'—H28'	120.0
H19A—C19—H19B	109.5	C29'—C28'—H28'	120.0
C21—O20—C2	110.60 (13)	C30'—C29'—C28'	120.18 (19)
O23—C21—O20	124.81 (17)	C30'—C29'—H29'	119.9
O23—C21—O22	123.59 (16)	C28'—C29'—H29'	119.9
O20—C21—O22	111.60 (15)	C31'—C30'—C29'	120.00 (19)
C21—O22—C1	111.40 (13)	C31'—C30'—H30'	120.0
C25—O24—C7	116.45 (13)	C29'—C30'—H30'	120.0
O26—C25—O24	123.80 (16)	C30'—C31'—C32'	120.44 (18)
O26—C25—C27	123.31 (16)	C30'—C31'—H31'	119.8
O24—C25—C27	112.90 (15)	C32'—C31'—H31'	119.8
C32—C27—C28	119.41 (16)	C31'—C32'—C27'	119.73 (17)
C32—C27—C25	122.92 (16)	C31'—C32'—H32'	120.1
C28—C27—C25	117.67 (16)	C27'—C32'—H32'	120.1
C29—C28—C27	120.35 (17)	C10'—O33'—H33'	109.5
C29—C28—H28	119.8	C35'—O34'—C4'	115.53 (14)
C27—C28—H28	119.8	O36'—C35'—O34'	114.07 (17)
C28—C29—C30	120.01 (18)	O36'—C35'—H35D	108.7
C28—C29—H29	120.0	O34'—C35'—H35D	108.7
C30—C29—H29	120.0	O36'—C35'—H35F	108.7
C29—C30—C31	120.18 (17)	O34'—C35'—H35F	108.7
C29—C30—H30	119.9	H35D—C35'—H35F	107.6
C31—C30—H30	119.9	C35'—O36'—C37'	112.82 (17)
C30—C31—C32	120.23 (17)	O36'—C37'—H37D	109.5
C30—C31—H31	119.9	O36'—C37'—H37E	109.5
C32—C31—H31	119.9	H37D—C37'—H37E	109.5
C31—C32—C27	119.80 (17)	O36'—C37'—H37F	109.5
C31—C32—H32	120.1	H37D—C37'—H37F	109.5
C27—C32—H32	120.1	H37E—C37'—H37F	109.5
C10—O33—H33	109.5	C2P—C1P—H1PA	109.5
C35—O34—C4	115.66 (13)	C2P—C1P—H1PB	109.5
O36—C35—O34	114.13 (14)	H1PA—C1P—H1PB	109.5
O36—C35—H35A	108.7	C2P—C1P—H1PC	109.5
O34—C35—H35A	108.7	H1PA—C1P—H1PC	109.5
O36—C35—H35B	108.7	H1PB—C1P—H1PC	109.5
O34—C35—H35B	108.7	C1P—C2P—C3P	113.0 (6)
H35A—C35—H35B	107.6	C1P—C2P—H2PA	109.0
C35—O36—C37	112.85 (15)	C3P—C2P—H2PA	109.0
O36—C37—H37B	109.5	C1P—C2P—H2PB	109.0
O36—C37—H37C	109.5	C3P—C2P—H2PB	109.0
H37B—C37—H37C	109.5	H2PA—C2P—H2PB	107.8
O36—C37—H37A	109.5	C4P—C3P—C2P	113.9 (5)
H37B—C37—H37A	109.5	C4P—C3P—H3PA	108.8
H37C—C37—H37A	109.5	C2P—C3P—H3PA	108.8
O22'—C1'—C14'	107.45 (13)	C4P—C3P—H3PB	108.8
O22'—C1'—C15'	109.95 (13)	C2P—C3P—H3PB	108.8
C14'—C1'—C15'	111.00 (14)	H3PA—C3P—H3PB	107.7
O22'—C1'—C2'	101.43 (13)	C3P—C4P—C5P	113.0 (6)
C14'—C1'—C2'	116.01 (14)	C3P—C4P—H4PA	109.0
C15'—C1'—C2'	110.45 (13)	C5P—C4P—H4PA	109.0
O20'—C2'—C3'	111.74 (13)	C3P—C4P—H4PB	109.0
O20'—C2'—C1'	104.56 (13)	C5P—C4P—H4PB	109.0
C3'—C2'—C1'	119.08 (14)	H4PA—C4P—H4PB	107.8
O20'—C2'—H2'	106.9	C4P—C5P—H5PA	109.5
C3'—C2'—H2'	106.9	C4P—C5P—H5PB	109.5
C1'—C2'—H2'	106.9	H5PA—C5P—H5PB	109.5
C4'—C3'—C2'	112.31 (14)	C4P—C5P—H5PC	109.5
C4'—C3'—C8'	115.02 (14)	H5PA—C5P—H5PC	109.5
C2'—C3'—C8'	108.62 (13)	H5PB—C5P—H5PC	109.5
			
O22—C1—C2—O20	6.62 (15)	C14'—C1'—C2'—O20'	−109.03 (15)
C14—C1—C2—O20	−108.85 (15)	C15'—C1'—C2'—O20'	123.57 (14)
C15—C1—C2—O20	123.41 (14)	O22'—C1'—C2'—C3'	132.64 (15)
O22—C1—C2—C3	132.30 (14)	C14'—C1'—C2'—C3'	16.6 (2)
C14—C1—C2—C3	16.8 (2)	C15'—C1'—C2'—C3'	−110.81 (17)
C15—C1—C2—C3	−110.91 (17)	O20'—C2'—C3'—C4'	13.44 (19)
O20—C2—C3—C4	11.23 (19)	C1'—C2'—C3'—C4'	−108.67 (17)
C1—C2—C3—C4	−110.86 (16)	O20'—C2'—C3'—C8'	−114.93 (15)
O20—C2—C3—C8	−116.69 (14)	C1'—C2'—C3'—C8'	122.97 (16)
C1—C2—C3—C8	121.21 (16)	C2'—C3'—C4'—O34'	62.79 (17)
C2—C3—C4—O34	61.23 (17)	C8'—C3'—C4'—O34'	−172.30 (13)
C8—C3—C4—O34	−173.60 (13)	C2'—C3'—C4'—C5'	−176.44 (15)
C2—C3—C4—C5	−178.60 (14)	C8'—C3'—C4'—C5'	−51.5 (2)
C8—C3—C4—C5	−53.42 (19)	O34'—C4'—C5'—C6'	172.16 (14)
O34—C4—C5—C6	173.67 (14)	C3'—C4'—C5'—C6'	55.1 (2)
C3—C4—C5—C6	56.31 (19)	C4'—C5'—C6'—C7'	−59.6 (2)
C4—C5—C6—C7	−59.01 (19)	C5'—C6'—C7'—O24'	−177.36 (14)
C5—C6—C7—O24	−179.24 (13)	C5'—C6'—C7'—C8'	61.9 (2)
C5—C6—C7—C8	60.47 (19)	O24'—C7'—C8'—C19'	−54.39 (18)
O24—C7—C8—C19	−54.43 (17)	C6'—C7'—C8'—C19'	67.17 (19)
C6—C7—C8—C19	67.71 (18)	O24'—C7'—C8'—C9'	65.65 (16)
O24—C7—C8—C9	65.48 (16)	C6'—C7'—C8'—C9'	−172.78 (14)
C6—C7—C8—C9	−172.38 (14)	O24'—C7'—C8'—C3'	−176.55 (13)
O24—C7—C8—C3	−176.86 (13)	C6'—C7'—C8'—C3'	−54.99 (19)
C6—C7—C8—C3	−54.72 (18)	C4'—C3'—C8'—C19'	−71.54 (18)
C4—C3—C8—C19	−70.39 (18)	C2'—C3'—C8'—C19'	55.28 (18)
C2—C3—C8—C19	56.45 (18)	C4'—C3'—C8'—C7'	49.07 (19)
C4—C3—C8—C7	50.29 (18)	C2'—C3'—C8'—C7'	175.89 (14)
C2—C3—C8—C7	177.14 (13)	C4'—C3'—C8'—C9'	163.86 (14)
C4—C3—C8—C9	164.85 (14)	C2'—C3'—C8'—C9'	−69.33 (17)
C2—C3—C8—C9	−68.31 (17)	C19'—C8'—C9'—C10'	−66.3 (2)
C19—C8—C9—C10	−64.8 (2)	C7'—C8'—C9'—C10'	174.41 (15)
C7—C8—C9—C10	176.12 (14)	C3'—C8'—C9'—C10'	59.1 (2)
C3—C8—C9—C10	60.8 (2)	C8'—C9'—C10'—O33'	161.84 (15)
C8—C9—C10—O33	155.68 (15)	C8'—C9'—C10'—C11'	−82.4 (2)
C8—C9—C10—C11	−87.7 (2)	O33'—C10'—C11'—C12'	89.06 (16)
O33—C10—C11—C12	95.01 (16)	C9'—C10'—C11'—C12'	−28.5 (2)
C9—C10—C11—C12	−24.3 (2)	O33'—C10'—C11'—C15'	−138.00 (15)
O33—C10—C11—C15	−132.92 (15)	C9'—C10'—C11'—C15'	104.46 (18)
C9—C10—C11—C15	107.80 (18)	C15'—C11'—C12'—C13'	−21.8 (2)
C15—C11—C12—C13	−22.0 (2)	C10'—C11'—C12'—C13'	115.02 (18)
C10—C11—C12—C13	113.52 (18)	C15'—C11'—C12'—C18'	155.27 (15)
C15—C11—C12—C18	155.30 (15)	C10'—C11'—C12'—C18'	−67.92 (19)
C10—C11—C12—C18	−69.16 (19)	C18'—C12'—C13'—C14'	−177.07 (16)
C18—C12—C13—C14	−177.24 (16)	C11'—C12'—C13'—C14'	−0.2 (3)
C11—C12—C13—C14	0.0 (3)	C12'—C13'—C14'—C1'	−10.8 (2)
C12—C13—C14—C1	−11.1 (2)	O22'—C1'—C14'—C13'	164.26 (14)
O22—C1—C14—C13	164.41 (13)	C15'—C1'—C14'—C13'	44.01 (19)
C15—C1—C14—C13	44.66 (19)	C2'—C1'—C14'—C13'	−83.12 (18)
C2—C1—C14—C13	−83.13 (18)	O22'—C1'—C15'—C16'	51.20 (18)
O22—C1—C15—C16	52.28 (18)	C14'—C1'—C15'—C16'	169.96 (14)
C14—C1—C15—C16	170.27 (14)	C2'—C1'—C15'—C16'	−59.92 (18)
C2—C1—C15—C16	−59.38 (18)	O22'—C1'—C15'—C17'	−66.92 (18)
O22—C1—C15—C17	−66.29 (17)	C14'—C1'—C15'—C17'	51.83 (18)
C14—C1—C15—C17	51.70 (18)	C2'—C1'—C15'—C17'	−178.04 (14)
C2—C1—C15—C17	−177.95 (14)	O22'—C1'—C15'—C11'	175.93 (13)
O22—C1—C15—C11	176.05 (12)	C14'—C1'—C15'—C11'	−65.31 (17)
C14—C1—C15—C11	−65.96 (17)	C2'—C1'—C15'—C11'	64.81 (17)
C2—C1—C15—C11	64.39 (17)	C12'—C11'—C15'—C16'	176.85 (14)
C12—C11—C15—C16	176.78 (14)	C10'—C11'—C15'—C16'	43.1 (2)
C10—C11—C15—C16	43.7 (2)	C12'—C11'—C15'—C1'	52.47 (17)
C12—C11—C15—C1	52.87 (17)	C10'—C11'—C15'—C1'	−81.28 (18)
C10—C11—C15—C1	−80.24 (17)	C12'—C11'—C15'—C17'	−67.12 (17)
C12—C11—C15—C17	−66.44 (17)	C10'—C11'—C15'—C17'	159.12 (14)
C10—C11—C15—C17	160.45 (14)	C3'—C2'—O20'—C21'	−136.82 (14)
C3—C2—O20—C21	−135.34 (14)	C1'—C2'—O20'—C21'	−6.71 (17)
C1—C2—O20—C21	−4.71 (17)	C2'—O20'—C21'—O23'	−176.50 (16)
C2—O20—C21—O23	−179.45 (17)	C2'—O20'—C21'—O22'	3.45 (19)
C2—O20—C21—O22	0.49 (18)	O23'—C21'—O22'—C1'	−178.39 (16)
O23—C21—O22—C1	−175.69 (16)	O20'—C21'—O22'—C1'	1.65 (19)
O20—C21—O22—C1	4.37 (19)	C14'—C1'—O22'—C21'	116.66 (15)
C14—C1—O22—C21	115.03 (15)	C15'—C1'—O22'—C21'	−122.43 (15)
C15—C1—O22—C21	−124.50 (14)	C2'—C1'—O22'—C21'	−5.52 (17)
C2—C1—O22—C21	−6.83 (17)	C6'—C7'—O24'—C25'	91.63 (18)
C6—C7—O24—C25	88.35 (17)	C8'—C7'—O24'—C25'	−143.73 (15)
C8—C7—O24—C25	−146.75 (14)	C7'—O24'—C25'—O26'	2.1 (3)
C7—O24—C25—O26	−5.8 (2)	C7'—O24'—C25'—C27'	−178.74 (14)
C7—O24—C25—C27	173.98 (13)	O26'—C25'—C27'—C28'	9.8 (3)
O26—C25—C27—C32	−171.09 (17)	O24'—C25'—C27'—C28'	−169.38 (16)
O24—C25—C27—C32	9.1 (2)	O26'—C25'—C27'—C32'	−170.79 (18)
O26—C25—C27—C28	9.3 (3)	O24'—C25'—C27'—C32'	10.0 (2)
O24—C25—C27—C28	−170.50 (15)	C32'—C27'—C28'—C29'	−0.9 (3)
C32—C27—C28—C29	0.5 (3)	C25'—C27'—C28'—C29'	178.55 (18)
C25—C27—C28—C29	−179.89 (16)	C27'—C28'—C29'—C30'	0.7 (3)
C27—C28—C29—C30	0.4 (3)	C28'—C29'—C30'—C31'	0.3 (3)
C28—C29—C30—C31	−0.5 (3)	C29'—C30'—C31'—C32'	−1.2 (3)
C29—C30—C31—C32	−0.4 (3)	C30'—C31'—C32'—C27'	1.0 (3)
C30—C31—C32—C27	1.2 (3)	C28'—C27'—C32'—C31'	0.0 (3)
C28—C27—C32—C31	−1.3 (3)	C25'—C27'—C32'—C31'	−179.37 (16)
C25—C27—C32—C31	179.11 (16)	C5'—C4'—O34'—C35'	83.68 (19)
C5—C4—O34—C35	99.01 (17)	C3'—C4'—O34'—C35'	−155.31 (15)
C3—C4—O34—C35	−140.30 (14)	C4'—O34'—C35'—O36'	−79.7 (2)
C4—O34—C35—O36	−79.12 (18)	O34'—C35'—O36'—C37'	−66.5 (2)
O34—C35—O36—C37	−71.1 (2)	C1P—C2P—C3P—C4P	175.9 (6)
O22'—C1'—C2'—O20'	7.02 (15)	C2P—C3P—C4P—C5P	179.1 (10)

### (A) (±)-(1SR,5SR,6SR,7SR,10SR,11SR,13RS,14SR)-13-Hydroxy-7-methoxymethoxy-11,15,18,18-tetramethyl-3-oxo-2,4-dioxatetracyclo[12.3.1.01,5.06,11]octadec-15-en-10-yl benzoate pentane 0.25-solvate Hydrogen-bond geometry (Å, º)

*Cg*1 is the centroid of the C27–C32 benzene ring.

**Table:**

*D*—H···*A*	*D*—H	H···*A*	*D*···*A*	*D*—H···*A*
C14—H14*A*···O34	0.99	2.47	3.320 (2)	143
C14′—H14*D*···O34′	0.99	2.36	3.221 (2)	145
O33—H33···O22′^i^	0.84	2.05	2.8563 (17)	160
O33′—H33′···O22^ii^	0.84	2.05	2.8839 (16)	169
C7—H7···O26^iii^	1.00	2.28	3.236 (2)	159
C4′—H4′···O36^iv^	1.00	2.40	3.311 (2)	151
C17′—H17*F*···O33^i^	0.98	2.48	3.431 (2)	164
C30′—H30′···O23′^v^	0.95	2.53	3.453 (2)	163
C3*P*—H3*PB*···O33	0.99	2.49	3.369 (9)	149
C4*P*—H4*PA*···O33^i^	0.99	2.41	3.291 (12)	148
C16′—H16*D*···*Cg*1^i^	0.98	2.85	3.5315 (19)	127

Symmetry codes: (i) −*x*+1, −*y*+2, −*z*+1; (ii) −*x*+1, −*y*+1, −*z*+1; (iii) −*x*+1, −*y*+2, −*z*+2; (iv) *x*+1, *y*, *z*−1; (v) *x*, *y*−1, *z*.

### (B) (±)-(1SR,5SR,6SR,7SR,10SR,11SR,13SR,14SR)-13-Hydroxy-7-methoxymethoxy-11,15,18,18-tetramethyl-3-oxo-2,4-dioxatetracyclo[12.3.1.01,5.06,11]octadec-15-en-10-yl benzoate Crystal data

**Table:**

C_29_H_38_O_8_	*D*_x_ = 1.338 Mg m^−^^3^
*M**_r_* = 514.59	Melting point: 489.2 K
Monoclinic, *P*2_1_/*n*	Mo *K*α radiation, λ = 0.71073 Å
*a* = 9.3612 (6) Å	Cell parameters from 4716 reflections
*b* = 19.6336 (15) Å	θ = 2.4–23.9°
*c* = 14.1965 (9) Å	µ = 0.10 mm^−^^1^
β = 101.762 (2)°	*T* = 90 K
*V* = 2554.4 (3) Å^3^	Prism, colorless
*Z* = 4	0.23 × 0.23 × 0.14 mm
*F*(000) = 1104	

### (B) (±)-(1SR,5SR,6SR,7SR,10SR,11SR,13SR,14SR)-13-Hydroxy-7-methoxymethoxy-11,15,18,18-tetramethyl-3-oxo-2,4-dioxatetracyclo[12.3.1.01,5.06,11]octadec-15-en-10-yl benzoate Data collection

**Table:**

Bruker D8 Venture diffractometer	4480 independent reflections
Radiation source: fine-focus sealed tube	3212 reflections with *I* > 2σ(*I*)
Multilayered confocal mirror monochromator	*R*_int_ = 0.058
Detector resolution: 8.333 pixels mm^-1^	θ_max_ = 25.0°, θ_min_ = 2.5°
φ and ω scans	*h* = −11→11
Absorption correction: multi-scan (*SADABS*; Bruker, 2014)	*k* = −23→21
*T*_min_ = 0.98, *T*_max_ = 0.99	*l* = −16→16
23252 measured reflections	

### (B) (±)-(1SR,5SR,6SR,7SR,10SR,11SR,13SR,14SR)-13-Hydroxy-7-methoxymethoxy-11,15,18,18-tetramethyl-3-oxo-2,4-dioxatetracyclo[12.3.1.01,5.06,11]octadec-15-en-10-yl benzoate Refinement

**Table:**

Refinement on *F*^2^	Primary atom site location: structure-invariant direct methods
Least-squares matrix: full	Secondary atom site location: difference Fourier map
*R*[*F*^2^ > 2σ(*F*^2^)] = 0.043	Hydrogen site location: inferred from neighbouring sites
*wR*(*F*^2^) = 0.111	H-atom parameters constrained
*S* = 1.02	*w* = 1/[σ^2^(*F*_o_^2^) + (0.054*P*)^2^ + 1.0752*P*] where *P* = (*F*_o_^2^ + 2*F*_c_^2^)/3
4480 reflections	(Δ/σ)_max_ = 0.001
340 parameters	Δρ_max_ = 0.51 e Å^−^^3^
0 restraints	Δρ_min_ = −0.21 e Å^−^^3^

### (B) (±)-(1SR,5SR,6SR,7SR,10SR,11SR,13SR,14SR)-13-Hydroxy-7-methoxymethoxy-11,15,18,18-tetramethyl-3-oxo-2,4-dioxatetracyclo[12.3.1.01,5.06,11]octadec-15-en-10-yl benzoate Special details

**Table:**

Experimental. *M*.p. 488.2–489.2 K (not corrected); IR (film) 3483, 2940, 1799, 1717, 1274, 1099, 1042, 773 cm^-1^; ^1^H NMR (500 MHz, CDCl_3_, at 333 K) *δ* (p.p.m.) 8.02 (d, *J* = 7.7 Hz, 2H), 7.58 (t, *J* = 7.5 Hz, 1H), 7.46 (t, *J* = 7.7 Hz, 2H), 5.50 (s, 1H), 4.92 (d, *J* = 4.3 Hz, 1H), 4.91 (dd *J* = 11.7, 4.9 Hz, 1H), 4.80 (d, *J* = 6.9 Hz, 1H), 4.64 (d, *J* = 6.9 Hz, 1H), 4.31 (d, *J* = 10.9 Hz, 1H), 3.68 (ddd, *J* = 10.9, 10.6, 4.9 Hz, 1H), 3.39 (s, 3H), 3.19 (d, *J* = 16.9 Hz, 1H), 2.42 (dd, *J* = 10.9, 4.3 Hz, 1H), 2.33 (s, 1H), 2.33–2.27 (m, 1H), 2.26 (d, *J* = 16.9 Hz, 1H), 2.02 (dd, *J* = 16.0, 10.9 Hz, 1H), 1.95–1.87 (m, 1H), 1.88 (s, 3H), 1.82 (dddd, *J* = 13.6, 13.6, 13.6, 4.6 Hz, 1H), 1.72 (d, *J* = 16.0 Hz, 1H), 1.49–1.40 (m, 1H), 1.46 (s, 1H), 1.41 (s, 3H), 1.20 (s, 3H), 1.18 (s, 3H); ^13^C NMR (125 MHz, CDCl_3_, at 333 K) *δ* (p.p.m.) 166.3 (C), 154.4 (C), 136.3 (C), 133.4 (CH), 130.4 (C), 129.8 (CH), 128.7 (CH), 122.3 (CH), 97.7 (CH_2_), 88.5 (C), 79.5 (CH), 75.2 (CH), 74.3 (CH), 70.3 (CH), 60.0 (CH), 56.1 (CH_3_), 44.8 (CH_2_), 44.2 (CH), 42.0 (C), 39.0 (C), 32.0 (CH_2_), 30.7 (CH_2_), 28.6 (CH_3_), 25.6 (CH_2_), 22.9 (CH_3_), 20.8 (CH_3_), 16.1 (CH_3_); LRMS (EI) *m/z* 514 (*M*^+^, 3%), 483 (1), 469 (1), 453 (1), 409 (2), 393 (6), 348 (1), 332 (2), 121 (78), 105 (100), 77 (59); HRMS (EI) *m/z* calcd for C_29_H_38_O_8_^+^ [*M*]^+^ 514.2567, found 514.2560.
Geometry. All e.s.d.'s (except the e.s.d. in the dihedral angle between two l.s. planes) are estimated using the full covariance matrix. The cell e.s.d.'s are taken into account individually in the estimation of e.s.d.'s in distances, angles and torsion angles; correlations between e.s.d.'s in cell parameters are only used when they are defined by crystal symmetry. An approximate (isotropic) treatment of cell e.s.d.'s is used for estimating e.s.d.'s involving l.s. planes.
Refinement. Refinement of *F*^2^ against ALL reflections. The weighted *R*-factor *wR* and goodness of fit *S* are based on *F*^2^, conventional *R*-factors *R* are based on *F*, with *F* set to zero for negative *F*^2^. The threshold expression of *F*^2^ > σ(*F*^2^) is used only for calculating *R*-factors(gt) *etc*. and is not relevant to the choice of reflections for refinement. *R*-factors based on *F*^2^ are statistically about twice as large as those based on *F*, and *R*- factors based on ALL data will be even larger.Problematic one reflection with |*I*(obs)-*I*(calc)|/σW(*I*) greater than 10 (-1 0 1) has been omitted in the final refinement.

### (B) (±)-(1SR,5SR,6SR,7SR,10SR,11SR,13SR,14SR)-13-Hydroxy-7-methoxymethoxy-11,15,18,18-tetramethyl-3-oxo-2,4-dioxatetracyclo[12.3.1.01,5.06,11]octadec-15-en-10-yl benzoate Fractional atomic coordinates and isotropic or equivalent isotropic displacement parameters (Å^2^)

**Table:**

	*x*	*y*	*z*	*U*_iso_*/*U*_eq_	
C1	0.7169 (2)	0.18654 (10)	0.68618 (13)	0.0143 (4)	
C2	0.6544 (2)	0.17259 (10)	0.77738 (14)	0.0136 (4)	
H2	0.6782	0.2123	0.8195	0.016*	
C3	0.7047 (2)	0.10935 (10)	0.83870 (13)	0.0131 (4)	
H3	0.7884	0.0905	0.8158	0.016*	
C4	0.5888 (2)	0.05350 (10)	0.82692 (14)	0.0157 (5)	
H4	0.5005	0.0711	0.8454	0.019*	
C5	0.6453 (2)	−0.00784 (10)	0.88896 (14)	0.0181 (5)	
H5A	0.7279	−0.0275	0.8671	0.022*	
H5B	0.5695	−0.0422	0.8827	0.022*	
C6	0.6915 (2)	0.01330 (11)	0.99391 (14)	0.0192 (5)	
H6A	0.6073	0.0301	1.0169	0.023*	
H6B	0.7295	−0.026	1.0325	0.023*	
C7	0.8069 (2)	0.06816 (10)	1.00523 (13)	0.0155 (4)	
H7	0.8952	0.0491	0.9884	0.019*	
C8	0.7615 (2)	0.13268 (10)	0.94372 (14)	0.0135 (4)	
C9	0.9051 (2)	0.17425 (10)	0.94939 (14)	0.0164 (5)	
H9B	0.9393	0.1848	1.017	0.02*	
H9A	0.9752	0.1424	0.9326	0.02*	
C10	0.9238 (2)	0.24112 (10)	0.89436 (14)	0.0166 (5)	
H10	1.0181	0.259	0.9281	0.02*	
C11	0.9370 (2)	0.24049 (10)	0.78491 (14)	0.0172 (5)	
H11	1.0036	0.2782	0.7801	0.021*	
C12	1.0208 (2)	0.17776 (11)	0.76571 (14)	0.0174 (5)	
C13	0.9588 (2)	0.12865 (10)	0.70699 (14)	0.0167 (5)	
H13	1.0156	0.0911	0.6989	0.02*	
C14	0.8037 (2)	0.12964 (10)	0.65293 (14)	0.0154 (4)	
H14A	0.7581	0.0864	0.6617	0.018*	
H14B	0.8021	0.1351	0.5848	0.018*	
C15	0.8037 (2)	0.25338 (10)	0.69973 (14)	0.0154 (5)	
C16	0.7084 (2)	0.31455 (10)	0.71360 (14)	0.0191 (5)	
H16B	0.6447	0.3255	0.6535	0.029*	
H16C	0.7695	0.353	0.7355	0.029*	
H16A	0.6512	0.3035	0.7604	0.029*	
C17	0.8650 (2)	0.27030 (11)	0.60997 (15)	0.0209 (5)	
H17A	0.9377	0.2373	0.6029	0.031*	
H17B	0.908	0.3149	0.6168	0.031*	
H17C	0.7874	0.2693	0.5541	0.031*	
C18	1.1796 (2)	0.17407 (12)	0.81421 (16)	0.0242 (5)	
H18B	1.2193	0.1311	0.7998	0.036*	
H18C	1.1887	0.1783	0.8825	0.036*	
H18A	1.232	0.2104	0.7912	0.036*	
C19	0.6461 (2)	0.17224 (11)	0.98432 (14)	0.0176 (5)	
H19C	0.6149	0.2111	0.9444	0.026*	
H19A	0.6873	0.1871	1.0485	0.026*	
H19B	0.5639	0.1432	0.9854	0.026*	
O20	0.49732 (15)	0.17278 (7)	0.74303 (9)	0.0176 (3)	
C21	0.4646 (2)	0.18489 (10)	0.64927 (15)	0.0178 (5)	
O22	0.58327 (14)	0.19463 (7)	0.61213 (9)	0.0170 (3)	
O23	0.34190 (16)	0.18696 (8)	0.60281 (10)	0.0247 (4)	
O24	0.84116 (15)	0.09012 (7)	1.10583 (9)	0.0168 (3)	
C25	0.9381 (2)	0.05274 (10)	1.16731 (14)	0.0154 (4)	
O26	1.00007 (16)	0.00371 (7)	1.14311 (10)	0.0237 (4)	
C27	0.9612 (2)	0.07873 (10)	1.26731 (14)	0.0153 (4)	
C28	0.8754 (2)	0.13006 (11)	1.29508 (14)	0.0194 (5)	
H28	0.7979	0.1484	1.2508	0.023*	
C29	0.9062 (2)	0.15358 (12)	1.38896 (15)	0.0252 (5)	
H29	0.8491	0.1878	1.4077	0.03*	
C30	1.0214 (2)	0.12650 (11)	1.45491 (15)	0.0231 (5)	
H30	1.0417	0.1428	1.5178	0.028*	
C31	1.1063 (2)	0.07552 (11)	1.42812 (15)	0.0227 (5)	
H31	1.1832	0.0571	1.4728	0.027*	
C32	1.0765 (2)	0.05194 (10)	1.33430 (14)	0.0187 (5)	
H32	1.1342	0.0179	1.316	0.022*	
O33	0.82270 (15)	0.29278 (7)	0.90665 (10)	0.0200 (3)	
H33	0.8271	0.2993	0.9642	0.03*	
O34	0.55698 (14)	0.03544 (7)	0.72690 (9)	0.0188 (3)	
C35	0.4129 (2)	0.01081 (12)	0.69395 (15)	0.0232 (5)	
H35A	0.3957	−0.0281	0.7322	0.028*	
H35B	0.343	0.046	0.7007	0.028*	
O36	0.39527 (17)	−0.00779 (8)	0.59939 (11)	0.0289 (4)	
C37	0.4715 (3)	−0.06965 (12)	0.58715 (17)	0.0315 (6)	
H37C	0.5739	−0.0637	0.6126	0.047*	
H37A	0.4566	−0.0805	0.5199	0.047*	
H37B	0.4349	−0.1061	0.6207	0.047*	

### (B) (±)-(1SR,5SR,6SR,7SR,10SR,11SR,13SR,14SR)-13-Hydroxy-7-methoxymethoxy-11,15,18,18-tetramethyl-3-oxo-2,4-dioxatetracyclo[12.3.1.01,5.06,11]octadec-15-en-10-yl benzoate Atomic displacement parameters (Å^2^)

**Table:**

	*U*^11^	*U*^22^	*U*^33^	*U*^12^	*U*^13^	*U*^23^
C1	0.0139 (11)	0.0176 (11)	0.0096 (10)	0.0000 (9)	−0.0017 (8)	0.0003 (8)
C2	0.0092 (10)	0.0182 (11)	0.0126 (10)	0.0005 (8)	0.0003 (8)	−0.0006 (8)
C3	0.0116 (11)	0.0156 (10)	0.0126 (11)	0.0014 (8)	0.0039 (8)	−0.0003 (8)
C4	0.0154 (11)	0.0203 (11)	0.0114 (11)	−0.0014 (9)	0.0026 (9)	−0.0038 (8)
C5	0.0187 (11)	0.0147 (11)	0.0216 (12)	−0.0040 (9)	0.0060 (9)	−0.0018 (9)
C6	0.0218 (12)	0.0190 (11)	0.0174 (11)	−0.0008 (9)	0.0052 (9)	0.0041 (9)
C7	0.0192 (11)	0.0184 (11)	0.0091 (10)	0.0018 (9)	0.0030 (9)	−0.0003 (8)
C8	0.0132 (11)	0.0142 (11)	0.0129 (11)	0.0003 (8)	0.0025 (8)	−0.0007 (8)
C9	0.0167 (11)	0.0174 (11)	0.0133 (11)	0.0010 (9)	−0.0009 (9)	−0.0013 (8)
C10	0.0154 (11)	0.0171 (11)	0.0162 (11)	−0.0002 (9)	0.0010 (9)	−0.0025 (8)
C11	0.0153 (11)	0.0185 (11)	0.0179 (11)	−0.0039 (9)	0.0039 (9)	−0.0010 (9)
C12	0.0160 (11)	0.0205 (11)	0.0170 (11)	−0.0006 (9)	0.0067 (9)	−0.0004 (9)
C13	0.0176 (11)	0.0178 (11)	0.0162 (11)	0.0030 (9)	0.0071 (9)	0.0015 (9)
C14	0.0200 (11)	0.0156 (11)	0.0118 (11)	0.0001 (9)	0.0059 (9)	−0.0001 (8)
C15	0.0170 (11)	0.0153 (11)	0.0141 (11)	0.0000 (9)	0.0040 (9)	0.0005 (8)
C16	0.0217 (12)	0.0188 (11)	0.0152 (11)	0.0028 (9)	0.0003 (9)	0.0027 (8)
C17	0.0248 (12)	0.0187 (11)	0.0207 (12)	−0.0029 (9)	0.0079 (10)	0.0009 (9)
C18	0.0184 (12)	0.0282 (13)	0.0263 (13)	0.0004 (10)	0.0052 (10)	−0.0049 (10)
C19	0.0199 (12)	0.0188 (11)	0.0140 (11)	0.0016 (9)	0.0032 (9)	0.0003 (8)
O20	0.0137 (8)	0.0265 (8)	0.0119 (8)	0.0036 (6)	0.0011 (6)	0.0032 (6)
C21	0.0195 (13)	0.0173 (11)	0.0167 (12)	−0.0003 (9)	0.0041 (10)	0.0018 (9)
O22	0.0152 (8)	0.0225 (8)	0.0124 (7)	0.0000 (6)	0.0007 (6)	0.0024 (6)
O23	0.0157 (9)	0.0375 (10)	0.0189 (8)	0.0007 (7)	−0.0014 (7)	0.0077 (7)
O24	0.0197 (8)	0.0197 (8)	0.0101 (7)	0.0043 (6)	0.0011 (6)	0.0004 (6)
C25	0.0159 (11)	0.0149 (11)	0.0154 (11)	−0.0005 (9)	0.0033 (9)	0.0034 (8)
O26	0.0295 (9)	0.0225 (8)	0.0179 (8)	0.0102 (7)	0.0023 (7)	−0.0004 (6)
C27	0.0173 (11)	0.0139 (10)	0.0152 (11)	−0.0024 (9)	0.0042 (9)	0.0024 (8)
C28	0.0175 (12)	0.0242 (12)	0.0158 (12)	0.0022 (9)	0.0021 (9)	0.0018 (9)
C29	0.0241 (13)	0.0317 (13)	0.0203 (13)	0.0071 (10)	0.0060 (10)	−0.0027 (10)
C30	0.0268 (13)	0.0293 (13)	0.0135 (11)	−0.0049 (10)	0.0045 (10)	−0.0027 (9)
C31	0.0227 (12)	0.0235 (12)	0.0195 (12)	−0.0011 (10)	−0.0010 (10)	0.0050 (9)
C32	0.0221 (12)	0.0155 (11)	0.0181 (11)	0.0006 (9)	0.0031 (9)	0.0010 (9)
O33	0.0251 (8)	0.0187 (8)	0.0162 (8)	0.0041 (6)	0.0046 (7)	−0.0028 (6)
O34	0.0166 (8)	0.0248 (8)	0.0140 (8)	−0.0046 (6)	0.0004 (6)	−0.0050 (6)
C35	0.0183 (12)	0.0304 (13)	0.0196 (12)	−0.0042 (10)	0.0007 (9)	−0.0070 (9)
O36	0.0293 (9)	0.0288 (9)	0.0249 (9)	0.0012 (7)	−0.0031 (7)	−0.0049 (7)
C37	0.0328 (14)	0.0255 (13)	0.0355 (14)	−0.0026 (11)	0.0056 (11)	−0.0103 (11)

### (B) (±)-(1SR,5SR,6SR,7SR,10SR,11SR,13SR,14SR)-13-Hydroxy-7-methoxymethoxy-11,15,18,18-tetramethyl-3-oxo-2,4-dioxatetracyclo[12.3.1.01,5.06,11]octadec-15-en-10-yl benzoate Geometric parameters (Å, º)

**Table:**

C1—O22	1.469 (2)	C15—C17	1.536 (3)
C1—C14	1.512 (3)	C16—H16B	0.96
C1—C15	1.535 (3)	C16—H16C	0.96
C1—C2	1.550 (3)	C16—H16A	0.96
C2—O20	1.452 (2)	C17—H17A	0.96
C2—C3	1.534 (3)	C17—H17B	0.96
C2—H2	0.98	C17—H17C	0.96
C3—C4	1.528 (3)	C18—H18B	0.96
C3—C8	1.547 (3)	C18—H18C	0.96
C3—H3	0.98	C18—H18A	0.96
C4—O34	1.435 (2)	C19—H19C	0.96
C4—C5	1.522 (3)	C19—H19A	0.96
C4—H4	0.98	C19—H19B	0.96
C5—C6	1.522 (3)	O20—C21	1.325 (2)
C5—H5A	0.97	C21—O23	1.203 (2)
C5—H5B	0.97	C21—O22	1.337 (2)
C6—C7	1.511 (3)	O24—C25	1.342 (2)
C6—H6A	0.97	C25—O26	1.210 (2)
C6—H6B	0.97	C25—C27	1.482 (3)
C7—O24	1.464 (2)	C27—C32	1.389 (3)
C7—C8	1.548 (3)	C27—C28	1.395 (3)
C7—H7	0.98	C28—C29	1.384 (3)
C8—C19	1.535 (3)	C28—H28	0.93
C8—C9	1.561 (3)	C29—C30	1.382 (3)
C9—C10	1.556 (3)	C29—H29	0.93
C9—H9B	0.97	C30—C31	1.379 (3)
C9—H9A	0.97	C30—H30	0.93
C10—O33	1.422 (2)	C31—C32	1.384 (3)
C10—C11	1.584 (3)	C31—H31	0.93
C10—H10	0.98	C32—H32	0.93
C11—C12	1.515 (3)	O33—H33	0.82
C11—C15	1.571 (3)	O34—C35	1.419 (2)
C11—H11	0.98	C35—O36	1.368 (2)
C12—C13	1.328 (3)	C35—H35A	0.97
C12—C18	1.507 (3)	C35—H35B	0.97
C13—C14	1.498 (3)	O36—C37	1.437 (3)
C13—H13	0.93	C37—H37C	0.96
C14—H14A	0.97	C37—H37A	0.96
C14—H14B	0.97	C37—H37B	0.96
C15—C16	1.533 (3)		
			
O22—C1—C14	106.84 (15)	C13—C14—H14B	109.2
O22—C1—C15	110.43 (15)	C1—C14—H14B	109.2
C14—C1—C15	111.48 (16)	H14A—C14—H14B	107.9
O22—C1—C2	101.84 (15)	C16—C15—C1	112.24 (16)
C14—C1—C2	116.66 (16)	C16—C15—C17	105.23 (16)
C15—C1—C2	109.08 (15)	C1—C15—C17	111.01 (16)
O20—C2—C3	111.43 (15)	C16—C15—C11	114.78 (16)
O20—C2—C1	104.36 (14)	C1—C15—C11	106.00 (16)
C3—C2—C1	119.49 (16)	C17—C15—C11	107.56 (16)
O20—C2—H2	107.0	C15—C16—H16B	109.5
C3—C2—H2	107.0	C15—C16—H16C	109.5
C1—C2—H2	107.0	H16B—C16—H16C	109.5
C4—C3—C2	112.93 (16)	C15—C16—H16A	109.5
C4—C3—C8	114.52 (15)	H16B—C16—H16A	109.5
C2—C3—C8	108.22 (16)	H16C—C16—H16A	109.5
C4—C3—H3	106.9	C15—C17—H17A	109.5
C2—C3—H3	106.9	C15—C17—H17B	109.5
C8—C3—H3	106.9	H17A—C17—H17B	109.5
O34—C4—C5	110.78 (16)	C15—C17—H17C	109.5
O34—C4—C3	106.57 (15)	H17A—C17—H17C	109.5
C5—C4—C3	110.44 (16)	H17B—C17—H17C	109.5
O34—C4—H4	109.7	C12—C18—H18B	109.5
C5—C4—H4	109.7	C12—C18—H18C	109.5
C3—C4—H4	109.7	H18B—C18—H18C	109.5
C4—C5—C6	110.28 (17)	C12—C18—H18A	109.5
C4—C5—H5A	109.6	H18B—C18—H18A	109.5
C6—C5—H5A	109.6	H18C—C18—H18A	109.5
C4—C5—H5B	109.6	C8—C19—H19C	109.5
C6—C5—H5B	109.6	C8—C19—H19A	109.5
H5A—C5—H5B	108.1	H19C—C19—H19A	109.5
C7—C6—C5	110.65 (16)	C8—C19—H19B	109.5
C7—C6—H6A	109.5	H19C—C19—H19B	109.5
C5—C6—H6A	109.5	H19A—C19—H19B	109.5
C7—C6—H6B	109.5	C21—O20—C2	110.43 (15)
C5—C6—H6B	109.5	O23—C21—O20	123.82 (19)
H6A—C6—H6B	108.1	O23—C21—O22	123.79 (18)
O24—C7—C6	108.86 (15)	O20—C21—O22	112.39 (18)
O24—C7—C8	106.83 (15)	C21—O22—C1	110.93 (15)
C6—C7—C8	114.51 (17)	C25—O24—C7	117.26 (15)
O24—C7—H7	108.8	O26—C25—O24	123.40 (18)
C6—C7—H7	108.8	O26—C25—C27	124.13 (18)
C8—C7—H7	108.8	O24—C25—C27	112.46 (17)
C19—C8—C3	112.46 (16)	C32—C27—C28	119.50 (18)
C19—C8—C7	109.62 (16)	C32—C27—C25	117.84 (18)
C3—C8—C7	107.59 (15)	C28—C27—C25	122.63 (18)
C19—C8—C9	112.39 (16)	C29—C28—C27	119.61 (19)
C3—C8—C9	109.13 (15)	C29—C28—H28	120.2
C7—C8—C9	105.30 (16)	C27—C28—H28	120.2
C10—C9—C8	126.80 (17)	C30—C29—C28	120.3 (2)
C10—C9—H9B	105.6	C30—C29—H29	119.8
C8—C9—H9B	105.6	C28—C29—H29	119.8
C10—C9—H9A	105.6	C31—C30—C29	120.4 (2)
C8—C9—H9A	105.6	C31—C30—H30	119.8
H9B—C9—H9A	106.1	C29—C30—H30	119.8
O33—C10—C9	113.25 (16)	C30—C31—C32	119.6 (2)
O33—C10—C11	108.23 (15)	C30—C31—H31	120.2
C9—C10—C11	121.68 (16)	C32—C31—H31	120.2
O33—C10—H10	103.9	C31—C32—C27	120.6 (2)
C9—C10—H10	103.9	C31—C32—H32	119.7
C11—C10—H10	103.9	C27—C32—H32	119.7
C12—C11—C15	110.59 (16)	C10—O33—H33	109.5
C12—C11—C10	109.26 (16)	C35—O34—C4	113.70 (14)
C15—C11—C10	123.08 (16)	O36—C35—O34	109.31 (16)
C12—C11—H11	104.0	O36—C35—H35A	109.8
C15—C11—H11	104.0	O34—C35—H35A	109.8
C10—C11—H11	104.0	O36—C35—H35B	109.8
C13—C12—C18	121.05 (19)	O34—C35—H35B	109.8
C13—C12—C11	121.81 (19)	H35A—C35—H35B	108.3
C18—C12—C11	117.12 (18)	C35—O36—C37	112.38 (17)
C12—C13—C14	124.47 (19)	O36—C37—H37C	109.5
C12—C13—H13	117.8	O36—C37—H37A	109.5
C14—C13—H13	117.8	H37C—C37—H37A	109.5
C13—C14—C1	112.09 (16)	O36—C37—H37B	109.5
C13—C14—H14A	109.2	H37C—C37—H37B	109.5
C1—C14—H14A	109.2	H37A—C37—H37B	109.5
			
O22—C1—C2—O20	1.49 (18)	O22—C1—C14—C13	164.37 (15)
C14—C1—C2—O20	−114.39 (18)	C15—C1—C14—C13	43.6 (2)
C15—C1—C2—O20	118.23 (16)	C2—C1—C14—C13	−82.6 (2)
O22—C1—C2—C3	126.81 (17)	O22—C1—C15—C16	50.0 (2)
C14—C1—C2—C3	10.9 (3)	C14—C1—C15—C16	168.56 (16)
C15—C1—C2—C3	−116.45 (19)	C2—C1—C15—C16	−61.2 (2)
O20—C2—C3—C4	15.6 (2)	O22—C1—C15—C17	−67.5 (2)
C1—C2—C3—C4	−106.25 (19)	C14—C1—C15—C17	51.1 (2)
O20—C2—C3—C8	−112.22 (17)	C2—C1—C15—C17	−178.64 (16)
C1—C2—C3—C8	125.90 (18)	O22—C1—C15—C11	176.00 (14)
C2—C3—C4—O34	59.3 (2)	C14—C1—C15—C11	−65.40 (19)
C8—C3—C4—O34	−176.20 (15)	C2—C1—C15—C11	64.86 (19)
C2—C3—C4—C5	179.71 (16)	C12—C11—C15—C16	178.07 (16)
C8—C3—C4—C5	−55.8 (2)	C10—C11—C15—C16	46.4 (3)
O34—C4—C5—C6	174.93 (15)	C12—C11—C15—C1	53.6 (2)
C3—C4—C5—C6	57.1 (2)	C10—C11—C15—C1	−78.1 (2)
C4—C5—C6—C7	−57.8 (2)	C12—C11—C15—C17	−65.2 (2)
C5—C6—C7—O24	176.31 (15)	C10—C11—C15—C17	163.09 (17)
C5—C6—C7—C8	56.8 (2)	C3—C2—O20—C21	−130.71 (17)
C4—C3—C8—C19	−69.6 (2)	C1—C2—O20—C21	−0.4 (2)
C2—C3—C8—C19	57.3 (2)	C2—O20—C21—O23	179.11 (19)
C4—C3—C8—C7	51.2 (2)	C2—O20—C21—O22	−1.0 (2)
C2—C3—C8—C7	178.13 (15)	O23—C21—O22—C1	−178.01 (19)
C4—C3—C8—C9	164.96 (16)	O20—C21—O22—C1	2.1 (2)
C2—C3—C8—C9	−68.11 (19)	C14—C1—O22—C21	120.70 (17)
O24—C7—C8—C19	−49.8 (2)	C15—C1—O22—C21	−117.90 (17)
C6—C7—C8—C19	70.8 (2)	C2—C1—O22—C21	−2.14 (19)
O24—C7—C8—C3	−172.37 (14)	C6—C7—O24—C25	83.5 (2)
C6—C7—C8—C3	−51.8 (2)	C8—C7—O24—C25	−152.31 (16)
O24—C7—C8—C9	71.32 (18)	C7—O24—C25—O26	2.0 (3)
C6—C7—C8—C9	−168.07 (16)	C7—O24—C25—C27	−178.54 (15)
C19—C8—C9—C10	−65.5 (2)	O26—C25—C27—C32	10.3 (3)
C3—C8—C9—C10	60.0 (2)	O24—C25—C27—C32	−169.09 (17)
C7—C8—C9—C10	175.21 (18)	O26—C25—C27—C28	−171.9 (2)
C8—C9—C10—O33	52.8 (3)	O24—C25—C27—C28	8.7 (3)
C8—C9—C10—C11	−78.9 (3)	C32—C27—C28—C29	0.0 (3)
O33—C10—C11—C12	−169.30 (16)	C25—C27—C28—C29	−177.76 (19)
C9—C10—C11—C12	−35.5 (2)	C27—C28—C29—C30	0.1 (3)
O33—C10—C11—C15	−37.1 (2)	C28—C29—C30—C31	−0.4 (3)
C9—C10—C11—C15	96.7 (2)	C29—C30—C31—C32	0.6 (3)
C15—C11—C12—C13	−23.7 (3)	C30—C31—C32—C27	−0.6 (3)
C10—C11—C12—C13	114.8 (2)	C28—C27—C32—C31	0.3 (3)
C15—C11—C12—C18	154.73 (17)	C25—C27—C32—C31	178.12 (18)
C10—C11—C12—C18	−66.8 (2)	C5—C4—O34—C35	88.2 (2)
C18—C12—C13—C14	−177.43 (18)	C3—C4—O34—C35	−151.61 (17)
C11—C12—C13—C14	1.0 (3)	C4—O34—C35—O36	−177.35 (16)
C12—C13—C14—C1	−10.5 (3)	O34—C35—O36—C37	72.7 (2)

### (B) (±)-(1SR,5SR,6SR,7SR,10SR,11SR,13SR,14SR)-13-Hydroxy-7-methoxymethoxy-11,15,18,18-tetramethyl-3-oxo-2,4-dioxatetracyclo[12.3.1.01,5.06,11]octadec-15-en-10-yl benzoate Hydrogen-bond geometry (Å, º)

*Cg*2 is the centroid of the C27–C32 benzene ring.

**Table:**

*D*—H···*A*	*D*—H	H···*A*	*D*···*A*	*D*—H···*A*
C2—H2···O33	0.98	2.27	3.200 (2)	157
C14—H14*A*···O34	0.97	2.47	3.293 (2)	142
O33—H33···O23^i^	0.82	1.96	2.7823 (19)	179
C7—H7···O26^ii^	0.98	2.50	3.353 (2)	145
C16—H16*A*···*Cg*2^iii^	0.98	2.93	3.594 (2)	128

Symmetry codes: (i) *x*+1/2, −*y*+1/2, *z*+1/2; (ii) −*x*+2, −*y*, −*z*+2; (iii) *x*−3/2, −*y*−1/2, *z*−3/2.

### (C) (±)-(1SR,5SR,6SR,7SR,10SR,11SR,14SR)-7-Methoxymethoxy-11,15,18,18-tetramethyl-3,13-dioxo-2,4-dioxatetracyclo[12.3.1.01,5.06,11]octadec-15-en-10-yl benzoate Crystal data

**Table:**

C_29_H_36_O_8_	*D*_x_ = 1.357 Mg m^−^^3^
*M**_r_* = 512.59	Melting point: 512.2 K
Monoclinic, *P*2_1_/*c*	Mo *K*α radiation, λ = 0.71073 Å
*a* = 13.2416 (8) Å	Cell parameters from 6186 reflections
*b* = 13.1779 (8) Å	θ = 2.8–24.9°
*c* = 15.2428 (8) Å	µ = 0.10 mm^−^^1^
β = 109.387 (2)°	*T* = 90 K
*V* = 2509.0 (3) Å^3^	Plate, colorless
*Z* = 4	0.22 × 0.14 × 0.09 mm
*F*(000) = 1096	

### (C) (±)-(1SR,5SR,6SR,7SR,10SR,11SR,14SR)-7-Methoxymethoxy-11,15,18,18-tetramethyl-3,13-dioxo-2,4-dioxatetracyclo[12.3.1.01,5.06,11]octadec-15-en-10-yl benzoate Data collection

**Table:**

Bruker D8 Venture diffractometer	4395 independent reflections
Radiation source: fine-focus sealed tube	3050 reflections with *I* > 2σ(*I*)
Multilayered confocal mirror monochromator	*R*_int_ = 0.063
Detector resolution: 8.333 pixels mm^-1^	θ_max_ = 25.0°, θ_min_ = 2.4°
φ and ω scans	*h* = −15→14
Absorption correction: multi-scan (*SADABS*; Bruker, 2014)	*k* = −15→15
*T*_min_ = 0.92, *T*_max_ = 0.99	*l* = −16→18
22512 measured reflections	

### (C) (±)-(1SR,5SR,6SR,7SR,10SR,11SR,14SR)-7-Methoxymethoxy-11,15,18,18-tetramethyl-3,13-dioxo-2,4-dioxatetracyclo[12.3.1.01,5.06,11]octadec-15-en-10-yl benzoate Refinement

**Table:**

Refinement on *F*^2^	Primary atom site location: structure-invariant direct methods
Least-squares matrix: full	Secondary atom site location: difference Fourier map
*R*[*F*^2^ > 2σ(*F*^2^)] = 0.044	Hydrogen site location: inferred from neighbouring sites
*wR*(*F*^2^) = 0.105	H-atom parameters constrained
*S* = 0.96	*w* = 1/[σ^2^(*F*_o_^2^) + (0.0391*P*)^2^ + 2.2049*P*] where *P* = (*F*_o_^2^ + 2*F*_c_^2^)/3
4395 reflections	(Δ/σ)_max_ = 0.001
339 parameters	Δρ_max_ = 0.29 e Å^−^^3^
0 restraints	Δρ_min_ = −0.25 e Å^−^^3^

### (C) (±)-(1SR,5SR,6SR,7SR,10SR,11SR,14SR)-7-Methoxymethoxy-11,15,18,18-tetramethyl-3,13-dioxo-2,4-dioxatetracyclo[12.3.1.01,5.06,11]octadec-15-en-10-yl benzoate Special details

**Table:**

Experimental. *M*.p. 510.7–512.2 K (not corrected); IR (film) 2934, 1804, 1718, 1689, 1668, 1272, 1230, 1108, 1058, 732, 713 cm^-1^; ^1^H NMR (500 MHz, CDCl_3_) *δ* (p.p.m.) 8.05–8.98 (m, 2H), 7.62–7.56 (m, 1H), 7.49–7.43 (m, 2H), 5.73 (bs, 1H), 4.79 (d, *J* = 6.9 Hz, 1H), 4.76 (dd, *J* = 11.0, 4.0 Hz, 1H), 4.60 (d, *J* = 6.9 Hz, 1H), 4.22 (d, *J* = 3.4 Hz, 1H), 3.62 (ddd, *J* = 10.6, 10.3, 5.2 Hz, 1H), 3.37 (s, 3H), 3.00 (bd, *J* = 17.0 Hz, 1H), 2.61 (d, *J* = 10.9 Hz, 1H), 2.51 (d, *J* = 17.0 Hz, 1H), 2.47 (s, 1H), 2.42–2.35 (m, 1H), 2.33 (dd, *J* = 10.6, 3.4 Hz, 1H), 2.13 (d, *J* = 10.9 Hz, 1H), 1.95–1.88 (m, 1H), 1.71 (s, 3H), 1.71–1.52 (m, 2H), 1.27 (s, 3H), 1.17 (s, 3H), 1.16 (s, 3H); ^13^C NMR (125 MHz, CDCl_3_) *δ* (p.p.m.) 211.2 (C), 165.9 (C), 153.7 (C), 135.8 (C), 133.5 (CH), 129.8 (C), 129.7 (CH), 128.7 (CH), 122.6 (CH), 97.8 (CH_2_), 87.0 (C), 79.3 (CH), 79.0 (CH), 74.7 (CH), 65.7 (CH), 56.0 (CH_3_), 51.2 (CH_2_), 48.0 (CH), 47.1 (C), 44.1 (C), 31.9 (CH_2_), 31.2 (CH_2_), 25.1 (CH_3_), 24.6 (CH_3_), 24.6 (CH_2_), 19.3 (CH_3_), 12.3 (CH_3_); LRMS (EI) *m/z* 512 (*M*^+^, 5%), 346 (9), 302 (7), 121 (28), 105 (100), 77 (55); HRMS (EI) *m/z* calcd for C_29_H_36_O_8_^+^ [*M*]^+^ 512.2410, found 514.2408.
Geometry. All e.s.d.'s (except the e.s.d. in the dihedral angle between two l.s. planes) are estimated using the full covariance matrix. The cell e.s.d.'s are taken into account individually in the estimation of e.s.d.'s in distances, angles and torsion angles; correlations between e.s.d.'s in cell parameters are only used when they are defined by crystal symmetry. An approximate (isotropic) treatment of cell e.s.d.'s is used for estimating e.s.d.'s involving l.s. planes.
Refinement. Refinement of *F*^2^ against ALL reflections. The weighted *R*-factor *wR* and goodness of fit *S* are based on *F*^2^, conventional *R*-factors *R* are based on *F*, with *F* set to zero for negative *F*^2^. The threshold expression of *F*^2^ > σ(*F*^2^) is used only for calculating *R*-factors(gt) *etc*. and is not relevant to the choice of reflections for refinement. *R*-factors based on *F*^2^ are statistically about twice as large as those based on *F*, and *R*- factors based on ALL data will be even larger.Problematic two reflections with |*I*(obs)-*I*(calc)|/σW(*I*) greater than 10 (1 1 0 and 11 4 3) have been omitted in the final refinement.

### (C) (±)-(1SR,5SR,6SR,7SR,10SR,11SR,14SR)-7-Methoxymethoxy-11,15,18,18-tetramethyl-3,13-dioxo-2,4-dioxatetracyclo[12.3.1.01,5.06,11]octadec-15-en-10-yl benzoate Fractional atomic coordinates and isotropic or equivalent isotropic displacement parameters (Å^2^)

**Table:**

	*x*	*y*	*z*	*U*_iso_*/*U*_eq_	
C1	0.81842 (17)	0.32712 (17)	0.49210 (14)	0.0156 (5)	
C2	0.83265 (18)	0.43504 (17)	0.53612 (14)	0.0157 (5)	
H2	0.8939	0.4312	0.5959	0.019*	
C3	0.73832 (17)	0.48566 (16)	0.55835 (14)	0.0141 (5)	
H3	0.6787	0.435	0.5422	0.017*	
C4	0.69530 (17)	0.58009 (17)	0.49830 (14)	0.0151 (5)	
H4	0.7551	0.629	0.5048	0.018*	
C5	0.60735 (18)	0.63170 (17)	0.52421 (14)	0.0186 (5)	
H5A	0.543	0.5879	0.5055	0.022*	
H5B	0.5884	0.6962	0.4894	0.022*	
C6	0.63941 (18)	0.65384 (17)	0.62826 (14)	0.0191 (5)	
H6A	0.699	0.7034	0.6466	0.023*	
H6B	0.578	0.6834	0.6427	0.023*	
C7	0.67390 (18)	0.55614 (17)	0.68165 (13)	0.0152 (5)	
H7	0.6122	0.5078	0.6641	0.018*	
C8	0.77033 (17)	0.50500 (16)	0.66456 (14)	0.0144 (5)	
C9	0.78727 (18)	0.40213 (17)	0.71791 (14)	0.0170 (5)	
H9B	0.8022	0.4176	0.7846	0.02*	
H9A	0.7185	0.3648	0.6963	0.02*	
C10	0.87422 (19)	0.33035 (17)	0.71100 (14)	0.0176 (5)	
C11	0.84777 (17)	0.23989 (17)	0.64233 (14)	0.0169 (5)	
H11	0.8853	0.1799	0.6791	0.02*	
C12	0.72983 (18)	0.21357 (17)	0.60918 (15)	0.0170 (5)	
C13	0.66633 (18)	0.23789 (17)	0.52443 (15)	0.0179 (5)	
H13	0.5922	0.2228	0.5084	0.021*	
C14	0.70379 (17)	0.28774 (17)	0.45229 (14)	0.0175 (5)	
H14A	0.6555	0.3451	0.4243	0.021*	
H14B	0.6994	0.2383	0.4023	0.021*	
C15	0.89166 (17)	0.24989 (17)	0.55936 (14)	0.0177 (5)	
C16	1.00975 (18)	0.28215 (18)	0.59015 (16)	0.0209 (5)	
H16A	1.0171	0.3507	0.6165	0.031*	
H16B	1.0346	0.2816	0.5363	0.031*	
H16C	1.0528	0.2347	0.6373	0.031*	
C17	0.88509 (19)	0.14530 (17)	0.51289 (16)	0.0221 (6)	
H17C	0.9039	0.152	0.4562	0.033*	
H17A	0.8121	0.1188	0.497	0.033*	
H17B	0.9351	0.0985	0.5559	0.033*	
C18	0.69208 (19)	0.15857 (18)	0.67848 (15)	0.0222 (6)	
H18B	0.6144	0.1488	0.6528	0.033*	
H18C	0.7096	0.1985	0.7358	0.033*	
H18A	0.7275	0.0924	0.6922	0.033*	
C19	0.87058 (18)	0.57156 (17)	0.70192 (15)	0.0186 (5)	
H19A	0.8809	0.5905	0.7665	0.028*	
H19B	0.8617	0.633	0.6639	0.028*	
H19C	0.9332	0.5337	0.6992	0.028*	
O20	0.86805 (11)	0.49630 (11)	0.47260 (9)	0.0165 (4)	
C21	0.87791 (17)	0.43902 (18)	0.40303 (15)	0.0169 (5)	
O22	0.85523 (12)	0.34137 (12)	0.41269 (10)	0.0187 (4)	
O23	0.90357 (12)	0.47131 (12)	0.34036 (10)	0.0224 (4)	
O24	0.70651 (11)	0.57550 (11)	0.78110 (9)	0.0170 (4)	
C25	0.63110 (18)	0.57567 (16)	0.82216 (15)	0.0146 (5)	
O26	0.53686 (12)	0.56418 (12)	0.78106 (10)	0.0203 (4)	
C27	0.67949 (18)	0.58868 (16)	0.92408 (14)	0.0150 (5)	
C28	0.61586 (19)	0.57355 (17)	0.97941 (15)	0.0212 (5)	
H28	0.5422	0.5574	0.9515	0.025*	
C29	0.6597 (2)	0.58201 (18)	1.07519 (15)	0.0237 (6)	
H29	0.6161	0.5712	1.1128	0.028*	
C30	0.7661 (2)	0.60592 (18)	1.11608 (16)	0.0255 (6)	
H30	0.796	0.6109	1.1818	0.031*	
C31	0.8296 (2)	0.62271 (18)	1.06145 (16)	0.0250 (6)	
H31	0.9028	0.6403	1.0896	0.03*	
C32	0.78646 (19)	0.61393 (18)	0.96603 (15)	0.0215 (5)	
H32	0.8304	0.6253	0.9288	0.026*	
O33	0.96560 (13)	0.34029 (12)	0.76362 (10)	0.0236 (4)	
O34	0.65380 (12)	0.54447 (11)	0.40409 (9)	0.0182 (4)	
C35	0.67590 (19)	0.60542 (18)	0.33721 (15)	0.0214 (6)	
H35A	0.7532	0.6218	0.3592	0.026*	
H35B	0.6603	0.566	0.2789	0.026*	
O36	0.61766 (12)	0.69614 (11)	0.31770 (10)	0.0200 (4)	
C37	0.50654 (18)	0.68122 (18)	0.27053 (16)	0.0243 (6)	
H37B	0.4746	0.647	0.312	0.036*	
H37C	0.4967	0.6393	0.2152	0.036*	
H37A	0.4717	0.7471	0.2518	0.036*	

### (C) (±)-(1SR,5SR,6SR,7SR,10SR,11SR,14SR)-7-Methoxymethoxy-11,15,18,18-tetramethyl-3,13-dioxo-2,4-dioxatetracyclo[12.3.1.01,5.06,11]octadec-15-en-10-yl benzoate Atomic displacement parameters (Å^2^)

**Table:**

	*U*^11^	*U*^22^	*U*^33^	*U*^12^	*U*^13^	*U*^23^
C1	0.0189 (13)	0.0175 (13)	0.0133 (11)	0.0015 (10)	0.0092 (10)	−0.0016 (9)
C2	0.0189 (13)	0.0148 (13)	0.0149 (11)	−0.0009 (10)	0.0079 (10)	0.0026 (9)
C3	0.0149 (12)	0.0126 (12)	0.0152 (11)	−0.0002 (10)	0.0056 (9)	−0.0016 (9)
C4	0.0166 (12)	0.0147 (13)	0.0130 (11)	−0.0004 (10)	0.0037 (10)	−0.0012 (9)
C5	0.0216 (13)	0.0163 (13)	0.0183 (11)	0.0057 (10)	0.0071 (10)	0.0028 (10)
C6	0.0228 (13)	0.0159 (13)	0.0212 (12)	0.0041 (10)	0.0109 (10)	−0.0006 (10)
C7	0.0192 (12)	0.0160 (13)	0.0112 (11)	−0.0035 (10)	0.0061 (10)	−0.0036 (9)
C8	0.0154 (12)	0.0156 (13)	0.0129 (11)	−0.0015 (10)	0.0058 (9)	−0.0010 (9)
C9	0.0199 (13)	0.0170 (13)	0.0150 (11)	0.0001 (10)	0.0070 (10)	−0.0003 (9)
C10	0.0209 (14)	0.0182 (13)	0.0137 (11)	−0.0007 (11)	0.0055 (11)	0.0052 (10)
C11	0.0187 (13)	0.0134 (13)	0.0178 (11)	0.0010 (10)	0.0049 (10)	0.0024 (9)
C12	0.0197 (13)	0.0128 (13)	0.0201 (12)	−0.0010 (10)	0.0089 (10)	−0.0034 (10)
C13	0.0180 (13)	0.0132 (13)	0.0226 (12)	−0.0012 (10)	0.0070 (10)	−0.0040 (10)
C14	0.0192 (13)	0.0151 (13)	0.0154 (11)	0.0009 (10)	0.0021 (10)	−0.0013 (9)
C15	0.0191 (13)	0.0144 (13)	0.0197 (12)	0.0023 (10)	0.0066 (10)	−0.0003 (10)
C16	0.0204 (13)	0.0179 (14)	0.0257 (12)	0.0051 (10)	0.0094 (10)	0.0026 (10)
C17	0.0260 (14)	0.0167 (14)	0.0252 (12)	0.0024 (11)	0.0106 (11)	−0.0014 (10)
C18	0.0242 (14)	0.0209 (14)	0.0229 (12)	−0.0011 (11)	0.0096 (11)	−0.0002 (10)
C19	0.0205 (13)	0.0180 (13)	0.0181 (11)	−0.0001 (10)	0.0074 (10)	−0.0028 (10)
O20	0.0199 (9)	0.0152 (9)	0.0177 (8)	−0.0015 (7)	0.0109 (7)	−0.0013 (7)
C21	0.0127 (12)	0.0184 (14)	0.0175 (12)	0.0024 (10)	0.0022 (10)	−0.0007 (10)
O22	0.0258 (9)	0.0165 (9)	0.0177 (8)	−0.0001 (7)	0.0122 (7)	−0.0014 (7)
O23	0.0254 (9)	0.0267 (10)	0.0201 (8)	−0.0039 (8)	0.0139 (7)	0.0004 (7)
O24	0.0194 (9)	0.0194 (9)	0.0139 (7)	−0.0010 (7)	0.0079 (7)	−0.0032 (6)
C25	0.0171 (13)	0.0079 (12)	0.0222 (12)	0.0014 (10)	0.0109 (11)	0.0004 (9)
O26	0.0175 (10)	0.0225 (10)	0.0211 (8)	−0.0019 (7)	0.0066 (7)	−0.0029 (7)
C27	0.0199 (13)	0.0108 (12)	0.0146 (11)	0.0022 (10)	0.0061 (10)	0.0003 (9)
C28	0.0256 (14)	0.0157 (13)	0.0235 (12)	0.0022 (11)	0.0098 (11)	0.0009 (10)
C29	0.0333 (16)	0.0241 (15)	0.0172 (12)	0.0041 (12)	0.0129 (12)	0.0027 (10)
C30	0.0390 (16)	0.0196 (14)	0.0150 (12)	0.0049 (12)	0.0052 (12)	0.0012 (10)
C31	0.0263 (14)	0.0230 (15)	0.0220 (13)	−0.0033 (11)	0.0029 (11)	−0.0035 (10)
C32	0.0243 (14)	0.0214 (14)	0.0212 (12)	−0.0008 (11)	0.0106 (11)	−0.0026 (10)
O33	0.0205 (10)	0.0264 (10)	0.0200 (8)	0.0017 (8)	0.0016 (8)	−0.0005 (7)
O34	0.0248 (9)	0.0179 (9)	0.0124 (7)	0.0017 (7)	0.0068 (7)	0.0018 (6)
C35	0.0241 (14)	0.0254 (15)	0.0162 (12)	0.0067 (11)	0.0087 (11)	0.0051 (10)
O36	0.0203 (9)	0.0162 (9)	0.0221 (8)	−0.0005 (7)	0.0052 (7)	0.0047 (7)
C37	0.0200 (14)	0.0243 (15)	0.0275 (13)	0.0014 (11)	0.0065 (11)	0.0056 (11)

### (C) (±)-(1SR,5SR,6SR,7SR,10SR,11SR,14SR)-7-Methoxymethoxy-11,15,18,18-tetramethyl-3,13-dioxo-2,4-dioxatetracyclo[12.3.1.01,5.06,11]octadec-15-en-10-yl benzoate Geometric parameters (Å, º)

**Table:**

C1—O22	1.459 (2)	C15—C17	1.539 (3)
C1—C14	1.526 (3)	C16—H16A	0.98
C1—C15	1.539 (3)	C16—H16B	0.98
C1—C2	1.557 (3)	C16—H16C	0.98
C2—O20	1.453 (2)	C17—H17C	0.98
C2—C3	1.550 (3)	C17—H17A	0.98
C2—H2	1.0	C17—H17B	0.98
C3—C4	1.538 (3)	C18—H18B	0.98
C3—C8	1.552 (3)	C18—H18C	0.98
C3—H3	1.0	C18—H18A	0.98
C4—O34	1.435 (2)	C19—H19A	0.98
C4—C5	1.510 (3)	C19—H19B	0.98
C4—H4	1.0	C19—H19C	0.98
C5—C6	1.528 (3)	O20—C21	1.343 (3)
C5—H5A	0.99	C21—O23	1.193 (2)
C5—H5B	0.99	C21—O22	1.341 (3)
C6—C7	1.510 (3)	O24—C25	1.343 (2)
C6—H6A	0.99	C25—O26	1.206 (3)
C6—H6B	0.99	C25—C27	1.481 (3)
C7—O24	1.454 (2)	C27—C32	1.387 (3)
C7—C8	1.540 (3)	C27—C28	1.390 (3)
C7—H7	1.0	C28—C29	1.385 (3)
C8—C19	1.534 (3)	C28—H28	0.95
C8—C9	1.558 (3)	C29—C30	1.374 (3)
C9—C10	1.521 (3)	C29—H29	0.95
C9—H9B	0.99	C30—C31	1.384 (3)
C9—H9A	0.99	C30—H30	0.95
C10—O33	1.217 (3)	C31—C32	1.380 (3)
C10—C11	1.548 (3)	C31—H31	0.95
C11—C12	1.514 (3)	C32—H32	0.95
C11—C15	1.563 (3)	O34—C35	1.403 (3)
C11—H11	1.0	C35—O36	1.400 (3)
C12—C13	1.326 (3)	C35—H35A	0.99
C12—C18	1.497 (3)	C35—H35B	0.99
C13—C14	1.500 (3)	O36—C37	1.421 (3)
C13—H13	0.95	C37—H37B	0.98
C14—H14A	0.99	C37—H37C	0.98
C14—H14B	0.99	C37—H37A	0.98
C15—C16	1.536 (3)		
			
O22—C1—C14	106.01 (16)	C1—C14—H14B	109.1
O22—C1—C15	109.12 (17)	H14A—C14—H14B	107.8
C14—C1—C15	111.33 (18)	C16—C15—C1	111.91 (18)
O22—C1—C2	102.36 (16)	C16—C15—C17	106.55 (18)
C14—C1—C2	116.36 (18)	C1—C15—C17	111.04 (18)
C15—C1—C2	110.96 (17)	C16—C15—C11	112.98 (17)
O20—C2—C3	111.62 (17)	C1—C15—C11	105.87 (17)
O20—C2—C1	103.89 (15)	C17—C15—C11	108.51 (18)
C3—C2—C1	119.76 (18)	C15—C16—H16A	109.5
O20—C2—H2	107.0	C15—C16—H16B	109.5
C3—C2—H2	107.0	H16A—C16—H16B	109.5
C1—C2—H2	107.0	C15—C16—H16C	109.5
C4—C3—C2	112.61 (17)	H16A—C16—H16C	109.5
C4—C3—C8	114.01 (17)	H16B—C16—H16C	109.5
C2—C3—C8	109.74 (17)	C15—C17—H17C	109.5
C4—C3—H3	106.7	C15—C17—H17A	109.5
C2—C3—H3	106.7	H17C—C17—H17A	109.5
C8—C3—H3	106.7	C15—C17—H17B	109.5
O34—C4—C5	109.67 (17)	H17C—C17—H17B	109.5
O34—C4—C3	105.90 (17)	H17A—C17—H17B	109.5
C5—C4—C3	111.98 (17)	C12—C18—H18B	109.5
O34—C4—H4	109.7	C12—C18—H18C	109.5
C5—C4—H4	109.7	H18B—C18—H18C	109.5
C3—C4—H4	109.7	C12—C18—H18A	109.5
C4—C5—C6	112.64 (18)	H18B—C18—H18A	109.5
C4—C5—H5A	109.1	H18C—C18—H18A	109.5
C6—C5—H5A	109.1	C8—C19—H19A	109.5
C4—C5—H5B	109.1	C8—C19—H19B	109.5
C6—C5—H5B	109.1	H19A—C19—H19B	109.5
H5A—C5—H5B	107.8	C8—C19—H19C	109.5
C7—C6—C5	109.07 (18)	H19A—C19—H19C	109.5
C7—C6—H6A	109.9	H19B—C19—H19C	109.5
C5—C6—H6A	109.9	C21—O20—C2	110.57 (17)
C7—C6—H6B	109.9	O23—C21—O22	124.3 (2)
C5—C6—H6B	109.9	O23—C21—O20	124.0 (2)
H6A—C6—H6B	108.3	O22—C21—O20	111.64 (18)
O24—C7—C6	110.13 (17)	C21—O22—C1	111.30 (16)
O24—C7—C8	106.08 (16)	C25—O24—C7	118.46 (17)
C6—C7—C8	113.65 (17)	O26—C25—O24	123.99 (19)
O24—C7—H7	109.0	O26—C25—C27	124.93 (19)
C6—C7—H7	109.0	O24—C25—C27	111.07 (19)
C8—C7—H7	109.0	C32—C27—C28	119.1 (2)
C19—C8—C7	110.53 (18)	C32—C27—C25	122.10 (19)
C19—C8—C3	112.58 (17)	C28—C27—C25	118.8 (2)
C7—C8—C3	107.06 (16)	C29—C28—C27	120.1 (2)
C19—C8—C9	110.29 (17)	C29—C28—H28	120.0
C7—C8—C9	106.03 (17)	C27—C28—H28	120.0
C3—C8—C9	110.09 (17)	C30—C29—C28	120.3 (2)
C10—C9—C8	119.09 (18)	C30—C29—H29	119.8
C10—C9—H9B	107.5	C28—C29—H29	119.8
C8—C9—H9B	107.5	C29—C30—C31	120.0 (2)
C10—C9—H9A	107.5	C29—C30—H30	120.0
C8—C9—H9A	107.5	C31—C30—H30	120.0
H9B—C9—H9A	107.0	C32—C31—C30	120.0 (2)
O33—C10—C9	119.8 (2)	C32—C31—H31	120.0
O33—C10—C11	118.8 (2)	C30—C31—H31	120.0
C9—C10—C11	121.29 (19)	C31—C32—C27	120.5 (2)
C12—C11—C10	112.27 (18)	C31—C32—H32	119.7
C12—C11—C15	111.64 (17)	C27—C32—H32	119.7
C10—C11—C15	114.89 (18)	C35—O34—C4	115.90 (17)
C12—C11—H11	105.7	O36—C35—O34	114.22 (17)
C10—C11—H11	105.7	O36—C35—H35A	108.7
C15—C11—H11	105.7	O34—C35—H35A	108.7
C13—C12—C18	123.5 (2)	O36—C35—H35B	108.7
C13—C12—C11	121.4 (2)	O34—C35—H35B	108.7
C18—C12—C11	115.12 (18)	H35A—C35—H35B	107.6
C12—C13—C14	124.3 (2)	C35—O36—C37	113.22 (18)
C12—C13—H13	117.9	O36—C37—H37B	109.5
C14—C13—H13	117.9	O36—C37—H37C	109.5
C13—C14—C1	112.63 (17)	H37B—C37—H37C	109.5
C13—C14—H14A	109.1	O36—C37—H37A	109.5
C1—C14—H14A	109.1	H37B—C37—H37A	109.5
C13—C14—H14B	109.1	H37C—C37—H37A	109.5
			
O22—C1—C2—O20	3.79 (19)	O22—C1—C14—C13	161.48 (17)
C14—C1—C2—O20	−111.28 (19)	C15—C1—C14—C13	42.9 (2)
C15—C1—C2—O20	120.08 (18)	C2—C1—C14—C13	−85.5 (2)
O22—C1—C2—C3	129.13 (18)	O22—C1—C15—C16	55.9 (2)
C14—C1—C2—C3	14.1 (3)	C14—C1—C15—C16	172.61 (17)
C15—C1—C2—C3	−114.6 (2)	C2—C1—C15—C16	−56.1 (2)
O20—C2—C3—C4	7.8 (2)	O22—C1—C15—C17	−63.0 (2)
C1—C2—C3—C4	−113.8 (2)	C14—C1—C15—C17	53.7 (2)
O20—C2—C3—C8	−120.39 (18)	C2—C1—C15—C17	−175.02 (17)
C1—C2—C3—C8	118.0 (2)	O22—C1—C15—C11	179.44 (16)
C2—C3—C4—O34	63.5 (2)	C14—C1—C15—C11	−63.9 (2)
C8—C3—C4—O34	−170.62 (16)	C2—C1—C15—C11	67.4 (2)
C2—C3—C4—C5	−177.00 (18)	C12—C11—C15—C16	176.93 (18)
C8—C3—C4—C5	−51.1 (2)	C10—C11—C15—C16	47.6 (3)
O34—C4—C5—C6	169.18 (18)	C12—C11—C15—C1	54.1 (2)
C3—C4—C5—C6	51.9 (3)	C10—C11—C15—C1	−75.2 (2)
C4—C5—C6—C7	−55.8 (2)	C12—C11—C15—C17	−65.1 (2)
C5—C6—C7—O24	179.09 (17)	C10—C11—C15—C17	165.54 (18)
C5—C6—C7—C8	60.2 (2)	C3—C2—O20—C21	−131.96 (18)
O24—C7—C8—C19	−55.8 (2)	C1—C2—O20—C21	−1.6 (2)
C6—C7—C8—C19	65.3 (2)	C2—O20—C21—O23	178.3 (2)
O24—C7—C8—C3	−178.79 (16)	C2—O20—C21—O22	−1.6 (2)
C6—C7—C8—C3	−57.6 (2)	O23—C21—O22—C1	−175.5 (2)
O24—C7—C8—C9	63.7 (2)	O20—C21—O22—C1	4.4 (2)
C6—C7—C8—C9	−175.17 (17)	C14—C1—O22—C21	117.38 (19)
C4—C3—C8—C19	−69.6 (2)	C15—C1—O22—C21	−122.62 (19)
C2—C3—C8—C19	57.8 (2)	C2—C1—O22—C21	−5.0 (2)
C4—C3—C8—C7	52.1 (2)	C6—C7—O24—C25	86.3 (2)
C2—C3—C8—C7	179.44 (17)	C8—C7—O24—C25	−150.29 (18)
C4—C3—C8—C9	166.90 (18)	C7—O24—C25—O26	−2.7 (3)
C2—C3—C8—C9	−65.7 (2)	C7—O24—C25—C27	175.64 (17)
C19—C8—C9—C10	−63.2 (2)	O26—C25—C27—C32	−171.9 (2)
C7—C8—C9—C10	177.06 (18)	O24—C25—C27—C32	9.8 (3)
C3—C8—C9—C10	61.6 (2)	O26—C25—C27—C28	9.1 (3)
C8—C9—C10—O33	86.1 (2)	O24—C25—C27—C28	−169.24 (19)
C8—C9—C10—C11	−98.0 (2)	C32—C27—C28—C29	−1.1 (3)
O33—C10—C11—C12	158.08 (19)	C25—C27—C28—C29	177.9 (2)
C9—C10—C11—C12	−17.9 (3)	C27—C28—C29—C30	0.4 (3)
O33—C10—C11—C15	−72.9 (3)	C28—C29—C30—C31	0.7 (4)
C9—C10—C11—C15	111.1 (2)	C29—C30—C31—C32	−1.0 (4)
C10—C11—C12—C13	104.9 (2)	C30—C31—C32—C27	0.2 (4)
C15—C11—C12—C13	−25.8 (3)	C28—C27—C32—C31	0.8 (3)
C10—C11—C12—C18	−75.4 (2)	C25—C27—C32—C31	−178.2 (2)
C15—C11—C12—C18	153.96 (19)	C5—C4—O34—C35	100.2 (2)
C18—C12—C13—C14	−176.4 (2)	C3—C4—O34—C35	−138.79 (18)
C11—C12—C13—C14	3.3 (3)	C4—O34—C35—O36	−72.6 (2)
C12—C13—C14—C1	−11.5 (3)	O34—C35—O36—C37	−68.7 (2)

### (C) (±)-(1SR,5SR,6SR,7SR,10SR,11SR,14SR)-7-Methoxymethoxy-11,15,18,18-tetramethyl-3,13-dioxo-2,4-dioxatetracyclo[12.3.1.01,5.06,11]octadec-15-en-10-yl benzoate Hydrogen-bond geometry (Å, º)

**Table:**

*D*—H···*A*	*D*—H	H···*A*	*D*···*A*	*D*—H···*A*
C14—H14*A*···O34	0.99	2.65	3.479 (3)	142
C31—H31···O33^i^	0.95	2.35	3.147 (3)	141
C19—H19*C*···O23^ii^	0.98	2.43	3.310 (3)	149
C16—H16*A*···O23^ii^	0.98	2.56	3.491 (3)	158

Symmetry codes: (i) −*x*+2, −*y*+1, −*z*+2; (ii) −*x*+2, −*y*+1, −*z*+1.
